# Niemann-Pick Type C Proteins Are Required for Sterol Transport and Appressorium-Mediated Plant Penetration of *Colletotrichum orbiculare*

**DOI:** 10.1128/mbio.02236-22

**Published:** 2022-09-26

**Authors:** Sayo Kodama, Naoki Kajikawa, Fumi Fukada, Yasuyuki Kubo

**Affiliations:** a Faculty of Agriculture, Setsunan University, Osaka, Japan; b Graduate School of Life and Environmental Sciences, Kyoto Prefectural University, Sakyo, Kyoto, Japan; University of Nebraska—Lincoln; Cornell University

**Keywords:** *Colletotrichum orbiculare*, fungal plant pathogen, Niemann-Pick type C proteins, appressorium penetration, sterol transport

## Abstract

Many biotrophic and hemibiotrophic fungal pathogens use appressoria to directly penetrate the host plant surface. In the cucumber anthracnose fungus *Colletotrichum orbiculare*, differentiation of appressoria requires a proper G_1_/S cell cycle progression, regulated by the GTPase-activating protein complex CoBub2-CoBfa1 and its downstream GTPase CoTem1. To explore the mechanisms by which the CoTem1 cascade regulates plant infection, we screened for CoTem1 interaction factors and identified a Niemann-Pick type C2 homolog (CoNpc2). Niemann-Pick type C proteins NPC1 and NPC2 are sterol-binding proteins required for sterol export from lysosomes (vacuoles) in humans and yeasts. We showed that CoNpc2 colocalized with CoNpc1 in late endosomes and vacuoles and that disruption of its gene resulted in aberrant sterol accumulation in vacuoles and loss of sterol membrane localization, indicating that NPC proteins are engaged in sterol transport in *C. orbiculare*. For appressorium infection, sterol transport and proper distribution mediated by CoNpc1 and CoNpc2 are critical for membrane integrity and membrane curvature with actin assembly, leading to penetration peg emergence and appressorial cone formation. Our results revealed a novel mechanism by which NPC proteins regulate appressorium-mediated plant infection.

## INTRODUCTION

Colletotrichum orbiculare is the causal fungus of anthracnose disease in cucurbits, including cucumbers, melons, and watermelons. Like many other *Colletotrichum* spp., *C. orbiculare* establishes a hemibiotrophic lifestyle (1). An infection structure called the appressorium mediates direct penetration of the host cuticle and epidermal cells using enormous turgor pressure accompanied by the secretion of cell wall-degrading enzymes. To convert the appressorial turgor pressure into mechanical force and rupture the leaf cuticle, the fungus develops a narrow penetration peg from the bottom of the appressorium. After appressorial penetration, the fungus first forms biotrophic infection vesicles and primary hyphae, and then it differentiates necrotrophic secondary hyphae, which kill and degrade host tissues.

Such infection-related morphogenesis is coupled to appropriate cell cycle progression and signal transduction (2). We previously demonstrated that *C. orbiculare* regulates proper cell cycle G_1_/S progression via the GTPase-activating protein (GAP) complex CoBub2-CoBfa1 and downstream GTPase CoTem1 to establish plant infection via the appressorium (3). Disturbance of either GAP component leads to earlier G_1_/S progression, resulting in earlier nuclear division and aberrant binucleation. GAP mutants do not form a penetration peg and cannot infect the plant and cause disease because the assembly of septin and actin at the appressorium pore is attenuated and the plant defense responses are enhanced. Although homologs of Bub2-Bfa1 and its GTPase Tem1 are highly conserved from yeasts to mammals, their functions vary depending on the species, such as mitotic exit, cytokinesis, or septum formation (4–6). Our comparative phenotypic analyses of mutants revealed that conservation of *BUB2* roles in cell cycle G_1_/S regulation, septum formation, and virulence in Colletotrichum higginsianum and Magnaporthe oryzae (7). In addition, CoTem1 regulates G_1_-to-S progression via CoBub2-CoBfa1 and negatively regulates septum formation in *C. orbiculare* (3). M. oryzae *SEP1*, a homologue of Schizosaccharomyces pombe Cdc7, a downstream target of Tem1/Spg1, coordinates nuclear division and negatively regulates cytokinesis (8). A conditional mutation in *SEP1* led to increased septation, multinucleation, and subsequent failure of appressorium-mediated infection. This phenotype is consistent with the CoTem1, whereas *TEM1* and *CDC7* homologs in fission yeast and other filamentous fungi positively regulate septum formation (9–11). These findings suggest that Bub2-Bfa1 and the Tem1 cascade are required for appressorium development in plant-pathogenic fungi. On the basis of this inference, we hypothesized that a specific protein interacts with CoTem1 and contributes to appressorium-mediated infection and pathogenicity.

Sterols are required for the organization and function of plasma membranes of eukaryotes, affecting membrane fluidity, integrity, and permeability (12–15). The main sterols in eukaryotes are represented by three predominant forms: cholesterol in vertebrates, phytosterols in plants, and ergosterol in fungi (16). Sterols dynamically move among organelles to maintain sterol homeostasis. In humans, Niemann-Pick type C proteins NPC1 and NPC2 bind cholesterol and are essential for lysosomal membrane integration before sterol redistribution to other cellular membranes (17–19). Genetic defects in NPC1 and NPC2 cause neurodegenerative disease as a result of cholesterol and other lipids accumulating in late endosomes and lysosomes (20). The molecular mechanism by which NPC proteins transport sterols has been extensively studied: NPC2, a soluble intralysosomal protein, binds a sterol molecule and is able to hand off the sterol to the lysosomal membrane protein NPC1, which then inserts the sterol into the lysosomal membrane (21, 22). The sterols are then transported to other organelles, including the endoplasmic reticulum (ER) and the plasma membrane, through mechanisms that have not yet been determined in detail (23, 24). In Saccharomyces cerevisiae, NPC proteins NCR1 and NPC2 play a role in sterol integration into the vacuolar membrane (25–28), but little attention has been paid to the function of NPC proteins in plant invasion by phytopathogenic fungi, including *C. orbiculare*.

Here, we used a yeast two-hybrid system to identify CoNpc2, a homolog of Niemann-Pick disease type C2 protein, as a CoTem1 physical interaction factor. CoNpc2 colocalized with a homolog of Niemann-Pick type C1 protein CoNpc1, and they have a critical role in the intracellular sterol transport of *C. orbiculare*. We demonstrate that appropriate sterol distribution is regulated by CoNpc1 and CoNpc2 and is required for membrane integrity and membrane curvature with actin assembly that leads to penetration peg emergence and the pathogenicity of *C. orbiculare*.

## RESULTS

### Identification of CoNpc2, a physical interactor of CoTem1.

To screen for the downstream factor that interacts with CoTem1 in the yeast two-hybrid (Y2H) assay, we mated the prey strains carrying the *C. orbiculare* cDNA library of vegetative mycelia and conidia and the bait strains carrying cDNA of CoTem1_96-302_ and grew them on the selective medium. Since the Y2H assay using full length of CoTem1 could not be evaluated properly due to autoactivation, we used CoTem1_96-302_ (3). This amino acid region contains the highly conserved Septum-promoting GTPase (Spg1) domain (see Fig. S1A in the supplemental material). Among 46 candidate genes that encoded products that interacted with CoTem1, we focused on 10 candidate interaction factors and named the genes *CPI1* to *CPI10* (CoTem1 Physical Interactor 1 to 10) (see Fig. S1B and Table S1 in the supplemental material).

10.1128/mbio.02236-22.1 FIG S1 Identification of physical interactor with CoTem_196-302_ using yeast two-hybrid assay. (A) CoTem1^T146A^ in which presumable effector binding domain (EBD) was mutated. (B) CPI1-CPI10 interact with CoTem_196-302_ in Y2H (Left side). CPI1-CPI10 were expressed in fusion with Gal4 DNA-binding domain (BD) protein and their interaction with CoTem_196-302_ fused with Gal4 activation domain (AD) were tested. Interaction was assessed from yeast growth on SD media lacking –Trp–Leu (DDO), and –Trp–Leu + X-α-Gal + AbA (DDOXA). Plasmids expressing the indicated proteins either as prey or as bait alone were used as negative controls and pGBKT7-53 (murine p53) and pGADT7-recT (SV40 large T antigen). (Right side) CPI2-CPI10 interact with CoTem1 at effector binding site. CPI1-CPI10 were expressed in fusion with Gal4 BD protein and their interaction with CoTem_196-302_^T146A^ fused with Gal4 AD were tested. (C) Pathogenicity assay of *Δcpi1*, *Δcpi2*, *Δcpi3*, *Δcpi5*, *Δcpi6*, *Δcpi8*, and *Δcpi10* mutants on intact cucumber cotyledons. The *Δcpi1*, *Δcpi3*, and *Δcpi6* mutants caused fewer and smaller lesions than did the wild type. Conidial suspensions were dropped onto cotyledons and incubated at 24°C for 5 days. (D) Mean lesion sizes (± SE; *n *=* *3) of detached cotyledons. Lesions of three leaves infected by each strain were measured at 5 days postinoculation in each replicate. ****, *P < *0.0001; **, *P < *0.01; ns, not significant (Student *t* test). Download FIG S1, TIF file, 1.7 MB. Copyright © 2022 Kodama et al. 2022 Kodama et al. https://creativecommons.org/licenses/by/4.0/ This content is distributed under the terms of the Creative Commons Attribution 4.0 International license.

10.1128/mbio.02236-22.9 TABLE S1 List of physical interaction factors with CoTem_196-302_ in a yeast two-hybrid assay. Download Table S1, DOCX file, 0.04 MB. Copyright © 2022 Kodama et al. 2022 Kodama et al. https://creativecommons.org/licenses/by/4.0/ This content is distributed under the terms of the Creative Commons Attribution 4.0 International license.

In a previous report, S. pombe Spg1, a homolog of CoTem1, interacted with downstream factor Cdc7 at the effector binding site. A mutation in this site resulted in a marked loss of specific interaction with Cdc7 (9). To test whether CoTem1 interacts with the 10 candidate interactors at the effector binding site, we generated CoTem1_T146A_ and performed the Y2H assay with these 10 genes (see Fig. S1A). CoTem1_T146A_ showed significant loss of interactions with 9 genes except *CPI1* (see Fig. S1B), suggesting that 9 genes are specific interactors with CoTem1 at the effector binding site.

**FIG 1:**
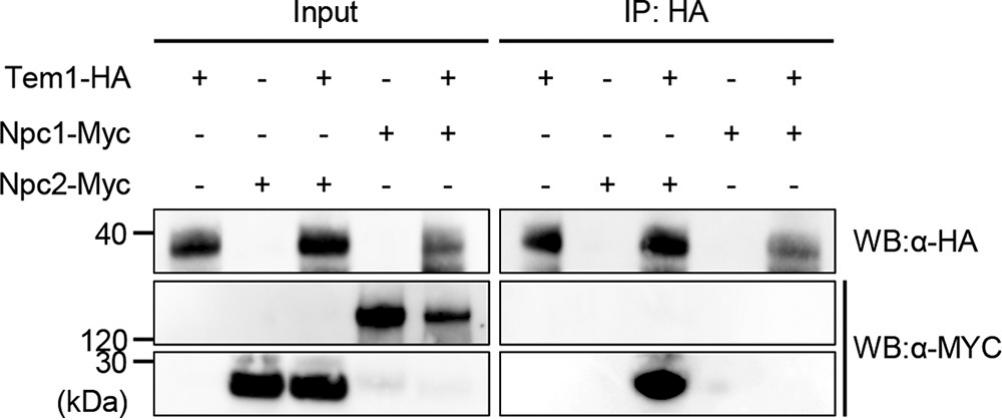
CoNpc2 interacts with CoTem1. The physical interaction of CoNpc2 and CoTem1 was demonstrated by Co-IP assays using protein from 4-day-old mycelia. CoTem1-HA (38 kDa) was immunoprecipitated with anti-HA magnetic beads. CoTem1-HA, CoNpc2-MYC (23 kDa), and CoNpc1-MYC (143 kDa) were detected by Western blotting (WB) with anti-HA or anti-MYC antibodies. The numbers on the left sides of the blots represent the marker size (kDa).

To test whether *CPI1* to *CPI10* are required for pathogenicity, we generated a deletion mutant for each gene and obtained *Δcpi1*, *Δcpi2*, *Δcpi3*, *Δcpi5*, *Δcpi6*, *Δcpi8*, and *Δcpi10* mutants. Then, cucumber cotyledons were inoculated with a conidial suspension of the respective deletion mutants. At 5 days postinoculation (dpi), the *Δcpi1*, *Δcpi3*, and *Δcpi6* mutants caused fewer and smaller lesions than did the wild type (see Fig. S1C and D). Since the lesions caused by the *Δcpi6* mutant were remarkably smaller than those caused by the *Δcpi1* and *Δcpi3* mutants, we focused on characterizing the function of *CPI6*. The *CPI6* gene (TDZ22676) is predicted to encode CoNpc2 based on 24 and 50% amino acid sequence identities to the sterol transporter Npc2 (Niemann Pick type C) of H. sapiens and S. cerevisiae, respectively. CoNpc2 contains a signal peptide and a MD-2-related lipid recognition (ML) domain (see Fig. S2A), and homologs are conserved among filamentous fungi, yeasts, and humans (see Fig. S2B). The physical interaction between CoTem1 and CoNpc2 was confirmed by a coimmunoprecipitation (Co-IP) assay (Fig. 1). Collectively, these results demonstrate that CoNpc2 is a physical interactor of CoTem1 that is required for the pathogenicity of *C. orbiculare*.

10.1128/mbio.02236-22.2 FIG S2 Diagrams and phylogenetic analyses of CoNpc2 and CoNpc1. (A) Diagram of CoNpc2. CoNpc2 putatively encodes 181 amino acids with candidate signal peptide (SP) and MD-2-related lipid-recognition (ML) domain. (B) Alignment of *Colletotrichum orbiculare* Npc2 with homologs from other filamentous ascomycetes (C.gl., *Colletotrichum gloeosporioides*; C.h., *Colletotrichum higginsianum*; C.gr., *Colletotrichum graminicola*; P.o., Pyricularia oryzae; N.c., Neurospora crassa; F.o., Fusarium oxysporum; F.g., Fusarium graminearum; A.n., Aspergillus nidulans; as well as S.c., Saccharomyces cerevisiae NPC2, and H.s., Homo sapiens NPC2). The alignment was constructed with ClustalW. Numbers on the right indicate amino acid residue positions. Conserved residues are highlighted with black, dark gray, and light gray represent 100, 80, and 70% amino acid conservation, respectively. The SP and ML domains are indicated by pink and orange lines, respectively. (C) Diagram of CoNpc1 encoding 1,271 amino acids. Pink box, signal peptide; light blue box, Niemann-Pick C type protein family domain (TIGR00917); green lines, transmembrane domain. (D) Phylogenetic tree of *Colletotrichum orbiculare* Npc1 (TDZ25151) with homologs of other filamentous ascomycetes (*Colletotrichum gloeosporioides* KAF3810155, *Colletotrichum higginsianum* TIC95183, *Colletotrichum graminicola* XP008098460, Pyricularia oryzae XP003718164, Neurospora crassa XP956086, Fusarium oxysporum EGU81378, Fusarium graminearum XP011328515, and Aspergillus nidulans XP659723), Saccharomyces cerevisiae NCR1 6R4L_A, and Homo sapiens NPC1 NP000262. The phylogenetic tree was constructed using MEGA-X with the minimum-evolution method and is based on 1,000 replicates. Bootstrap values are shown; the scale bar denotes evolutionary distances. Download FIG S2, TIF file, 0.7 MB. Copyright © 2022 Kodama et al. 2022 Kodama et al. https://creativecommons.org/licenses/by/4.0/ This content is distributed under the terms of the Creative Commons Attribution 4.0 International license.

### CoNpc2 colocalized with CoNpc1 in late endosomes and vacuoles.

In humans and yeasts, NPC2 functions with NPC1 (NCR1) in sterol transfer and sterol membrane integration (28–30). We identified the NPC1 homolog, CoNpc1 (TDZ25151), in a BLASTP search of the *C. orbiculare* genome. CoNpc1 shares 34 and 41% sequence identities with human NPC1 and yeast NCR1, respectively. CoNpc1 consists of a signal peptide, 13 transmembrane domains, and the Niemann-Pick C type protein family conserved domain similar to human NPC1 and yeast NCR1 (see Fig. S2C). In a phylogenetic tree based on the amino acid sequences, the NPC1 homologs from filamentous ascomycetes formed a single clade, in contrast to yeasts and humans (see Fig. S2D). In addition, CoNpc1 did not interact with CoTem1 in consistent with the Y2H screening results that CoNpc1 was not detected (Fig. 1).

**FIG 2:**
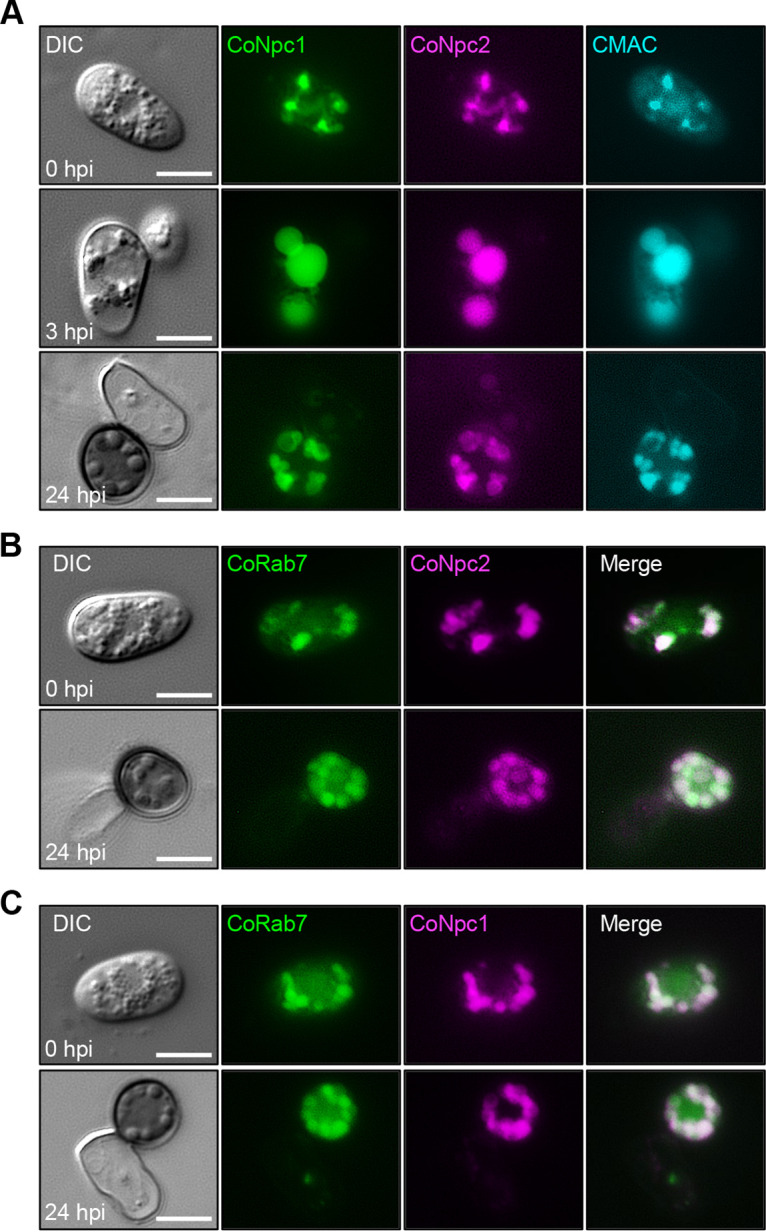
CoNpc2 colocalized with CoNpc1 and CoRab7 in vacuoles and granular bodies of conidia and appressoria of *C. orbiculare*. (A) Subcellular colocalization of CoNpc2-mCherry with CoNpc1-GFP in vacuoles during appressorial development. Conidial suspensions in distilled water were incubated on glass slides for 0, 3, and 24 h. The expression of CoNpc2-mCherry and CoNpc1-GFP was controlled under the native promoter. The vacuolar lumen was stained with 10 μM CMAC. Scale bar, 5 μm. (B and C) Localization of CoNpc2-mCherry (B), CoNpc1-mCherry (C), and CoRab7-GFP driven by the native promoter. Conidial suspensions in distilled water were incubated on glass slides for 0 and 24 h. Scale bar, 5 μm. The images are fluorescence micrographs.

To test whether CoNpc2 is involved in sterol transport with CoNpc1, we first used fluorescent protein tagging to localize the proteins in conidia and appressoria. CoNpc2-mCherry colocalized with CoNpc1-GFP at whole vacuoles stained with CMAC dye of conidia and mature appressoria (Fig. 2A; see also Fig. S3). CoNpc2-mCherry colocalized with a late endosome marker CoRab7-GFP in conidia and appressoria (Fig. 2B). Identically, CoNpc1-mCherry also colocalized with CoRab7-GFP (Fig. 2C). These observations are consistent with the localization of human Npc2 in late endosome and lysosomal compartments (19, 31, 32) and of yeast Npc2 in the vacuolar lumen (26). These results suggest that CoNpc2 and CoNpc1 function in late endosome and vacuoles.

10.1128/mbio.02236-22.3 FIG S3 Confirmation of no significant bleed-through between CoNpc1-GFP and CoNpc2-mCherry. Conidia of the respective single tagged strains CoNpc1-GFP and CoNpc2-mCherry were observed to measure bleed-through by imaging both channels under the same acquisition parameters. The expressions of CoNpc1-GFP and CoNpc2-mCherry were controlled under the native promoter. Scale bars, 5 μm. Images are fluorescence micrographs from a single focal plane. Download FIG S3, TIF file, 1.1 MB. Copyright © 2022 Kodama et al. 2022 Kodama et al. https://creativecommons.org/licenses/by/4.0/ This content is distributed under the terms of the Creative Commons Attribution 4.0 International license.

### CoNpc1 and CoNpc2 are required for intracellular sterol transport.

**FIG 3:**
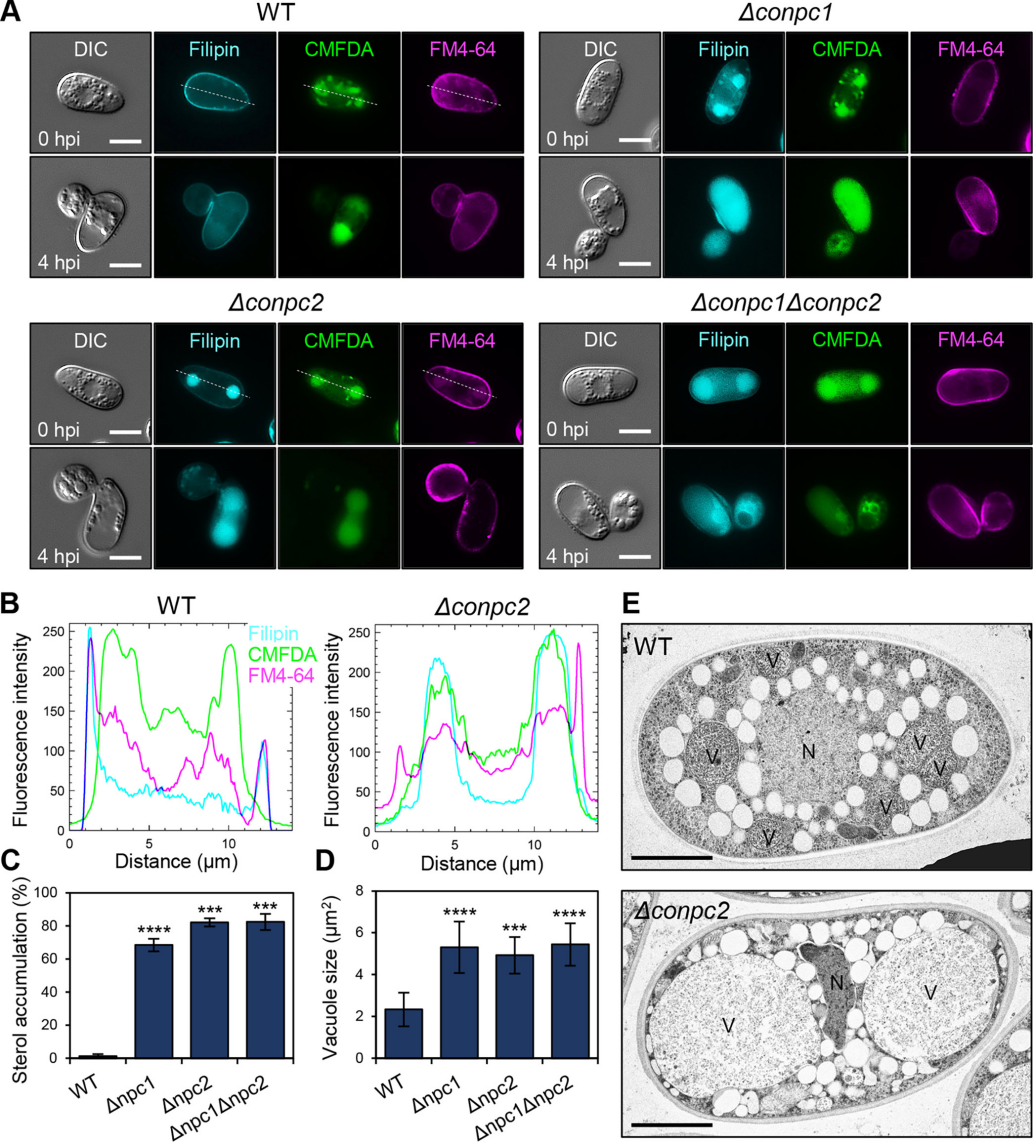
*CoNPC1* and *CoNPC2* contribute to sterol transport from vacuoles of *C. orbiculare*. (A) Localization of sterols in conidium and immature appressorium viewed using fluorescence microscopy. For sterol staining, a conidial suspension was incubated on a glass slide at 24°C, and then cells were stained at various times with 0.5 mg/mL filipin. The membrane and vacuolar lumen were stained with 8 μM FM4-64 and 10 μM CMFDA, respectively. Scale bar, 5 μm. (B) Fluorescence intensity profiles of filipin, FM4-64, and CMFDA along the line drawn across the conidium of the wild-type strain 104-T and the *Δconpc2* mutant in panel A. (C) Mean percentages (± the standard errors [SE; *n *=* *3]) of conidia with sterol accumulation in vacuoles of the indicated strains at 0 hpi. At least 100 conidia were scored for each replicate. Each strain was stained by filipin. **, *P < *0.01; ***, *P < *0.001 (Student *t* test). (D) Mean vacuole sizes (± SE; *n *=* *3) of conidia at 0 hpi. Each strain was strained with 10 μM CMFDA. At least 300 conidia were scored for each replicate. ***, *P < *0.001; ****, *P < *0.0001 (Student *t* test). (E) Conidia of the wild type (WT) and the *Δconpc2* mutant from 6-day-old PDA cultures imaged by TEM. N, nucleus, V, vacuole. Scale bars, 2 μm.

Human Niemann-Pick disease type C proteins (hNPC1 and hNPC2) bind cholesterol and are essential for cholesterol trafficking from the lysosome (17, 19, 33). Mutations in NPC genes cause abnormally high cholesterol accumulation in lysosomes (34). Similar to the human NPCs, the S. cerevisiae NPC proteins (NCR1 and NPC2) function as sterol transporters (28). To test whether CoNpc1 and CoNpc2 are involved in sterol transport, we looked for sterol accumulation in conidia and appressoria using filipin III staining during appressorium development. Sterol staining was observed along the conidial and appressorial membrane in the wild type, whereas filipin staining in *Δconpc1* and *Δconpc2* mutants was evident in the conidial vacuole at 0 to 4 h postinoculation (hpi) (Fig. 3A to C). In the conidia of *Δconpc1* and *Δconpc2* mutants, vacuolar lumen staining by CMFDA showed that the area of vacuoles was 2.1 to 2.4 times larger than in the wild type (Fig. 3A and D). We confirmed these results using transmission electron microscopy (TEM). Wild-type conidia contained multiple vacuoles less than 2 μm in diameter, whereas the *Δconpc2* mutant had two markedly larger vacuoles (Fig. 3E). Because the melanin layer in mature appressoria interfered with fluorescent staining using filipin, CMFDA and FM4-64, melanin biosynthesis was inhibited by carpropamid, a melanin inhibitor. At 24 hpi, the *Δconpc1* and *Δconpc2* mutants had fewer filipin-stained sterols at the plasma membrane in the conidium and mature appressorium compared to the wild type (see Fig. S4). These results indicate that CoNpc1 and CoNpc2 are engaged in sterol transport and proper sterol distribution in *C. orbiculare*. Although FM membrane marker dyes fluoresce intensely only when bound to membranes, at 24 hpi in the *Δconpc1* and *Δconpc2* mutants, FM4-64 fluoresced uniformly in the cytoplasm of the conidium and appressorium in contrast to the wild type (see Fig. S4). Considering the contribution of *CoNPC1* and *CoNPC2* to sterol transport, this finding suggests that deletion of these genes would affect membrane integrity.

10.1128/mbio.02236-22.4 FIG S4 *CoNPC1* and *CoNPC2* deletion impairs sterol localization at the plasma membrane of the mature appressorium. (A) For sterol staining, a conidial suspension was incubated on a glass slide at 24°C for 24 h, and then the cells were labeled with 0.5 mg/mL filipin. The membrane was stained with 8 μM FM4-64. +CP: to inhibit melanin biosynthesis, 1 μg/mL carpropamid was applied to conidial suspensions. Scale bar, 5 μm. (B) Fluorescence intensity profiles of both filipin and FM4-64 along the line drawn across appressoria of the wild type (WT) and the *Δconpc1 Δconpc2* mutant in panel A. Download FIG S4, TIF file, 2.9 MB. Copyright © 2022 Kodama et al. 2022 Kodama et al. https://creativecommons.org/licenses/by/4.0/ This content is distributed under the terms of the Creative Commons Attribution 4.0 International license.

Since sterol misdistribution in the conidium and appressorium of the *Δconpc1* and *Δconpc2* mutants could impair membrane integrity, we considered that deletion of these genes could be harmful for mycelial growth and different stress tolerance. The colony morphologies of the *Δconpc1* and *Δconpc2* mutants on different media were indistinguishable from that of the wild type (see Fig. S5A and B). The sporulation of mutants was similar to that of the wild type (see Fig. S5C). In addition, no significant differences in mycelial vacuolar morphology were observed between the *Δconpc1* and *Δconpc2* mutants and the wild type (see Fig. S5D). These findings suggest that *CoNPC1* and *CoNPC2* are not required for mycelial morphology and stress response during vegetative growth.

10.1128/mbio.02236-22.5 FIG S5 *CoNPC1* and *CoNPC2* is not required for mycelial growth, sporulation and morphogenesis of mycelial vacuole. (A) Mean colony diameter (± SE; *n *=* *3) on different culture media at 6 days post incubation (dpi). PDA (potato dextrose agar), CFW (PDA containing 100 μg/mL calcofluor white), SDS (PDA containing 0.01% SDS), CM (complete medium), and H_2_O_2_ (CM containing 2 mM H_2_O_2_). ns, not significant (Student *t* test). (B) Colony morphology on different culture media at 6 dpi. (C) Sporulation on PDA (± SE; *n *=* *3) at 6 dpi. ns, not significant (Student *t* test). (D) Vacuolar morphology of a mycelium. For vacuolar lumen staining, a conidial suspension was incubated in PSY (potato sucrose-yeast extract medium) at 28°C for 24 h, and then the cells were labeled with 8 μM FM4-64 and 10 μM CMFDA. Scale bars, 5 μm. Download FIG S5, TIF file, 2.4 MB. Copyright © 2022 Kodama et al. 2022 Kodama et al. https://creativecommons.org/licenses/by/4.0/ This content is distributed under the terms of the Creative Commons Attribution 4.0 International license.

### CoNpc2 is not involved in G_1_/S progression and nuclear autophagy.

Our previous study revealed that two-component GAP CoBub2-CoBfa1 regulates G_1_/S progression and autophagy via GTPase CoTem1 in *C. orbiculare*. Therefore, we hypothesized that CoNpc2, a physical interactor with CoTem1, is involved in cell cycle regulation. To test this hypothesis, we observed the nuclear division of the *Δconpc2* mutant during appressorium development. Our results showed that nuclear division at 6 hpi in the *Δconpc2* mutant appeared to be no different from that in the wild type (see Fig. S6A). Also, the deletion of *conpc2* did not significantly alter the phenotype of nucleus autophagy in conidia at 12 hpi (see Fig. S6B). These results indicate that *CoNPC2* is not involved in G_1_/S progression and nuclear autophagy.

10.1128/mbio.02236-22.6 FIG S6 *CoNPC2* is not involved in G_1_/S progression and nuclear autophagy. (A) Nuclear behavior in *Δconpc2* at 6 hpi. Mean percentage (±SE, *n *=* *3) of conidia and immature appressoria with single or multiple nuclei of the wild type and *Δconpc2* and *Δconpc2:CoNPC2* mutants at 6 hpi. Conidial suspension in distilled water was incubated on a slide glass at 24°C. At least 200 appressoria were scored. A two-tailed independent *t* test was used to compare values for WT and mutant strains (ns, not significant). (B) Mean percentage (± SE, *n *=* *3) of conidium and appressorium with single or multiple nuclei of the wild-type, *Δconpc2*, *Δconpc2:CoNPC2*, and *Δcotem1* strains at 12 hpi. The nucleus that remained in the conidium (type II) had been degraded by autophagy, while the appressorium retained its one nucleus (type I). Conidial suspension in distilled water was incubated on a glass slide at 24°C. At least 200 appressoria were scored. ns, not significant; **, *P < *0.01 (Student *t* test). Download FIG S6, TIF file, 0.2 MB. Copyright © 2022 Kodama et al. 2022 Kodama et al. https://creativecommons.org/licenses/by/4.0/ This content is distributed under the terms of the Creative Commons Attribution 4.0 International license.

To investigate the functional involvement of CoNpc2 with CoBub2-CoBfa1 and CoTem1 in sterol transport, we checked sterol accumulation in conidia using filipin staining. In the *Δcobub2*, *Δcobfa1*, and *Δcotem1* mutants, sterol was stained along the conidial membrane but not in vacuoles, similar to what was observed for the wild type (see Fig. S7). Therefore, CoBub2, CoBfa1, and CoTem1 do not seem to be involved in sterol transport in *C. orbiculare*.

10.1128/mbio.02236-22.7 FIG S7 CoBub2-CoBfa1 and CoTem1 are not involved in sterol transport. (A) Sterol accumulation in conidia of indicated strains viewed with fluorescent microscopy. For sterol staining, a conidial suspension was incubated on a slide glass at 24°C for 10 min, and then cells were labeled in 0.5 mg/mL filipin. Scale bars, 5 μm. (B) Mean percentages (± SE, *n *=* *3) of conidia with sterol accumulation in vacuoles of indicated strains at 10 min after incubation and stained with filipin. At least 100 conidia were scored. ns, not significant; ***, *P < *0.001 (Student *t* test). Download FIG S7, TIF file, 2.5 MB. Copyright © 2022 Kodama et al. 2022 Kodama et al. https://creativecommons.org/licenses/by/4.0/ This content is distributed under the terms of the Creative Commons Attribution 4.0 International license.

### *CoNPC1* and *CoNPC2* are essential for appressorium-mediated host cuticle penetration.

**FIG 4:**
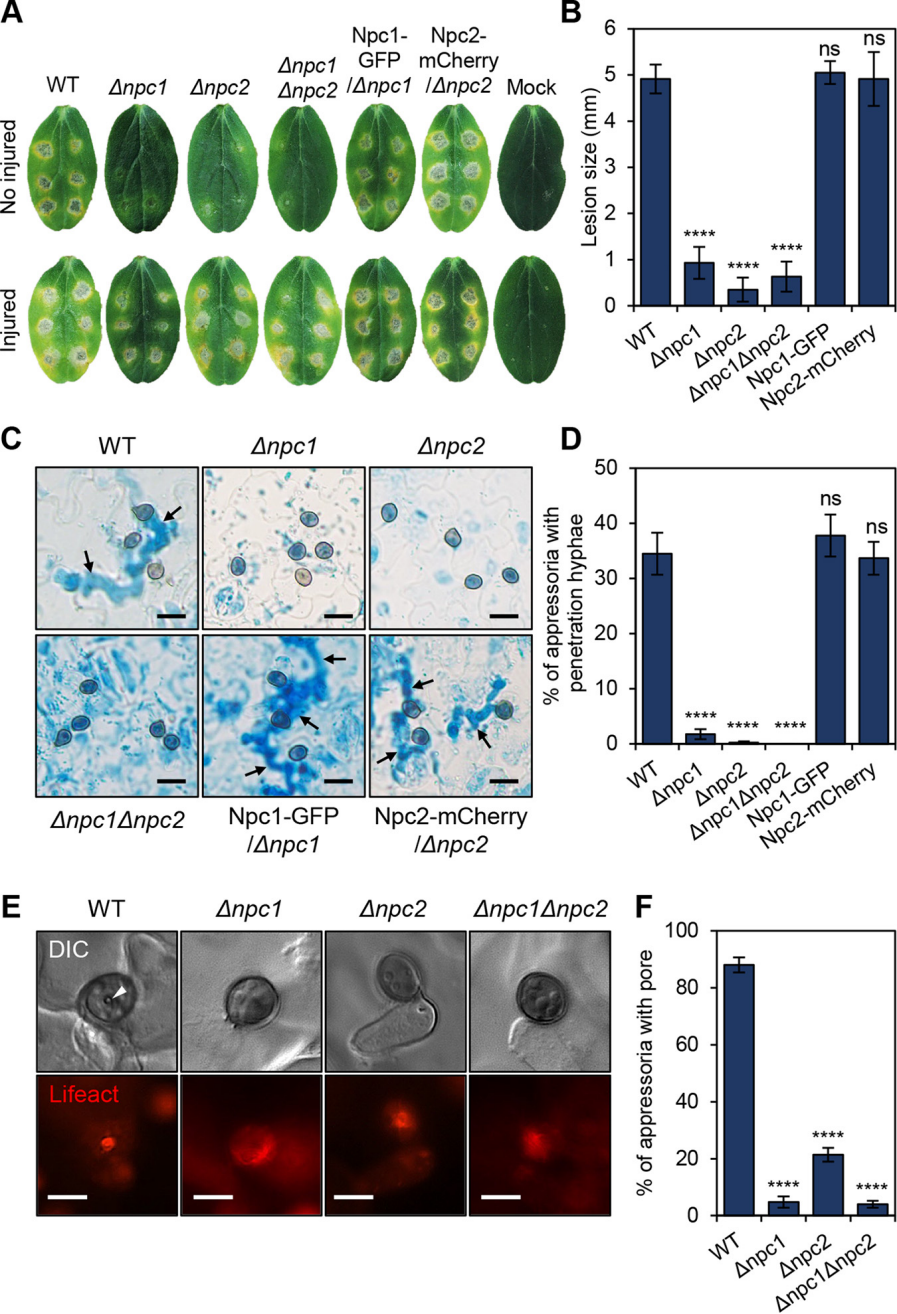
*CoNPC1* and *CoNPC2* are essential for appressorium-mediated penetration of the cuticle of cucumber cotyledons. (A) Pathogenicity of *Δconpc1* and *Δconpc2* mutants on detached cotyledons. Conidial suspension of strain was dropped onto unwounded or injured cotyledons and incubated at 24°C for 5 days. (B) Mean lesion size (± SE; *n *=* *3) of detached cotyledons. Lesions of five leaves infected by each strain were measured at 5 days postinoculation in each replicate. ****, *P < *0.0001; ns, not significant (Student *t* test). (C) Appressorial morphology and penetration ability of indicated strains after 72 h on detached cotyledons and viewed using bright-field microscopy. Arrows indicate penetration hyphae. Scale bars, 10 μm. (D) Mean percentage (± SE; *n *=* *3) of appressoria that formed penetration hyphae at 72 hpi. At least 300 appressoria were analyzed in each replicate. ****, *P < *0.0001; ns, not significant (Student *t* test). (E) Representative fluorescence micrographs showing actin assembly at the appressorial pore. Strains harboring LIFEACT-RFP were incubated on a cotyledon for 48 h. Arrowheads, appressorial pore. Scale bars, 5 μm. (F) Mean percentage (± SE; *n *=* *3) of appressoria that formed an appressorium pore after 48 h. At least 150 appressoria were scored in each replicate. ****, *P < *0.0001 (Student *t* test).

To analyze the function of *CoNPC1* and *CoNPC2* in infection-related morphogenesis, we examined appressorium formation on cucumber cotyledons. The *Δconpc1* and *Δconpc2* mutants formed morphologically normal appressoria, but almost no penetration hyphae were observed; thus, markedly fewer and smaller lesions formed on the host plant (Fig. 4A to D). Introducing CoNpc1-GFP into the *Δconpc1* mutant and CoNpc2-mCherry into the *Δconpc2* mutant restored appressorium penetration of the host and virulence. Thus, *Δconpc1* and *Δconpc2* mutants were functionally complemented by CoNpc1-GFP and CoNpc2-mCherry, respectively (Fig. 4A to D). The *Δconpc1* and *Δconpc2* mutants formed lesions similar to the wild type on wounded cucumber cotyledons (Fig. 4A), suggesting that *CoNPC1* and *CoNPC2* are not required for invasive growth. Furthermore, the mutants did not induce papillae in the non-host onion epidermis (see Fig. S8) and were defective in appressorium pore formation on the host plant surface (Fig. 4E and F). In addition, fluorescence of the F-actin marker Lifeact-RFP at the appressorium pore was diffused in *Δconpc1* and *Δconpc2* mutants compared to its localization in the wild type (Fig. 4E), suggesting that the assembly of actin at the appressorium pore was disturbed in these mutants. Therefore, *CoNPC1* and *CoNPC2* are required for appressorium-mediated host cuticle penetration.

10.1128/mbio.02236-22.8 FIG S8 *CoNPC1* and *CoNPC2* mutants did not induce papillae in non-host onion epidermis. (A) Representative images of papillae accumulation in the host cell below the appressorium at 72 hpi. Epidermis was inoculated with a conidial suspension of the wild-type 104-T or indicated mutant strains, then incubated for 24 to 72 h and observed with epi-fluorescence or bright-field microscopy. Scale bars = 20 μm. (B) Mean percentage of appressoria with papillae accumulation at each time (±SE; *n *=* *3). More than 200 appressoria were scored per replicate. Download FIG S8, TIF file, 2.9 MB. Copyright © 2022 Kodama et al. 2022 Kodama et al. https://creativecommons.org/licenses/by/4.0/ This content is distributed under the terms of the Creative Commons Attribution 4.0 International license.

**FIG 5:**
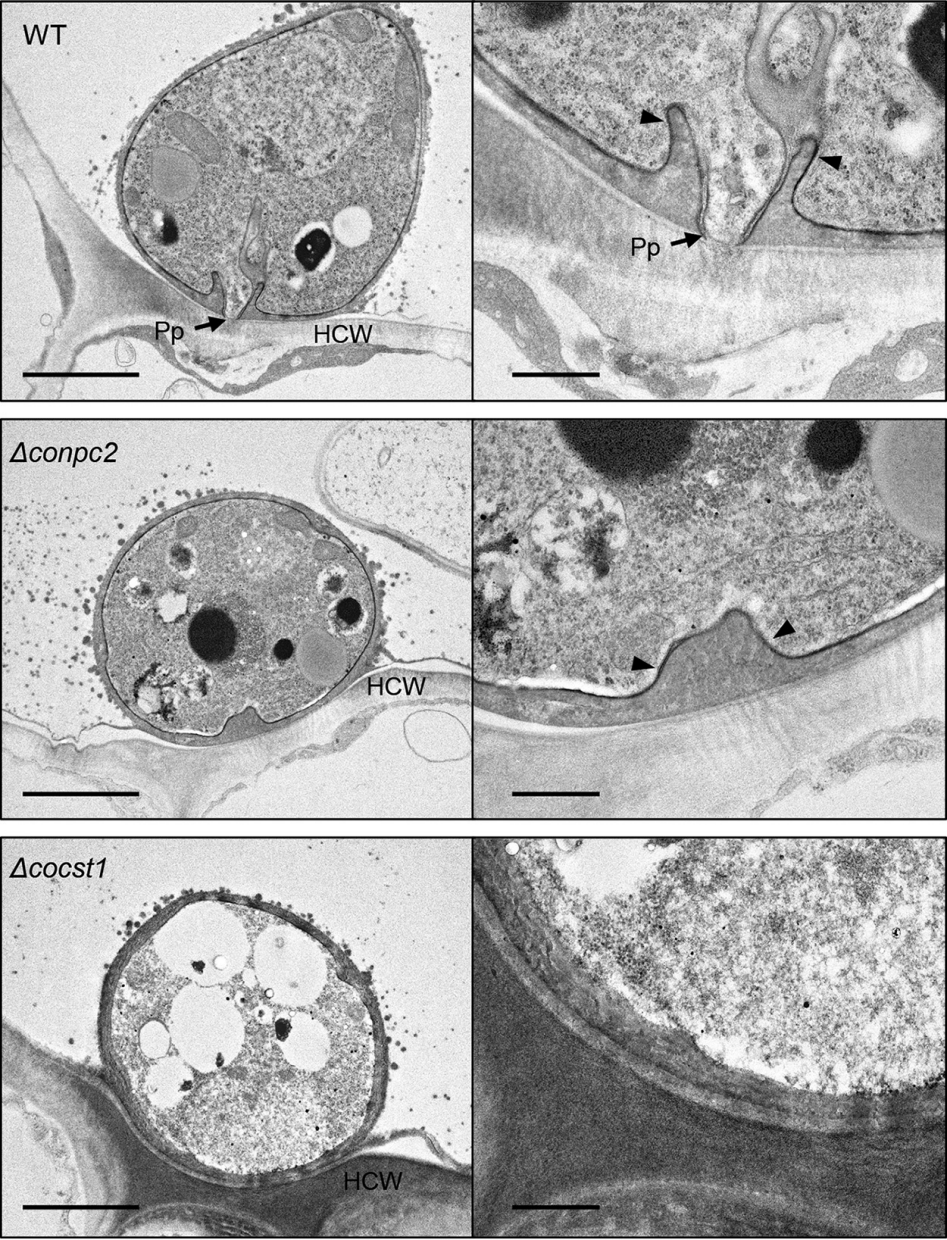
Immature appressorial cone of the *conpc2* mutant leads to defect in penetration peg formation. The appressoria of the wild-type strain 104-T and the *Δconpc2* and *Δcocst1* mutants on the abaxial surfaces of detached cucumber cotyledons after 48 h were observed using TEM. At least 20 appressoria were observed for each strain. Appressoria (Ap), penetration pegs (Pp), and host cell wall (Hcw) are indicated. Arrowheads, appressorial cone. Left scale bars, 2 μm; right scale bars, 500 nm.

To further investigate how the deletion of *CoNPC1* and *CoNPC2* causes defects in appressorium penetration, we examined the appressoria formed by the *Δconpc2* mutant using TEM. In the wild type, the appressoria formed a basal penetration peg surrounded by a funnel-shaped appressorial cone (35). However, the *Δconpc2* mutant showed defects in appressorial cone and peg formation (Fig. 5). The transcription factor gene *CoCST1* is downstream of mitogen-activated protein kinase pathway and is required for appressorium penetration (36). Unlike the wild type and the *Δconpc2* mutant, the *Δcocst1* mutant did not form any cones. Thus, the immature appressorial cone is a remarkable phenotype of the sterol transporter-deficient mutant.

**FIG 6:**
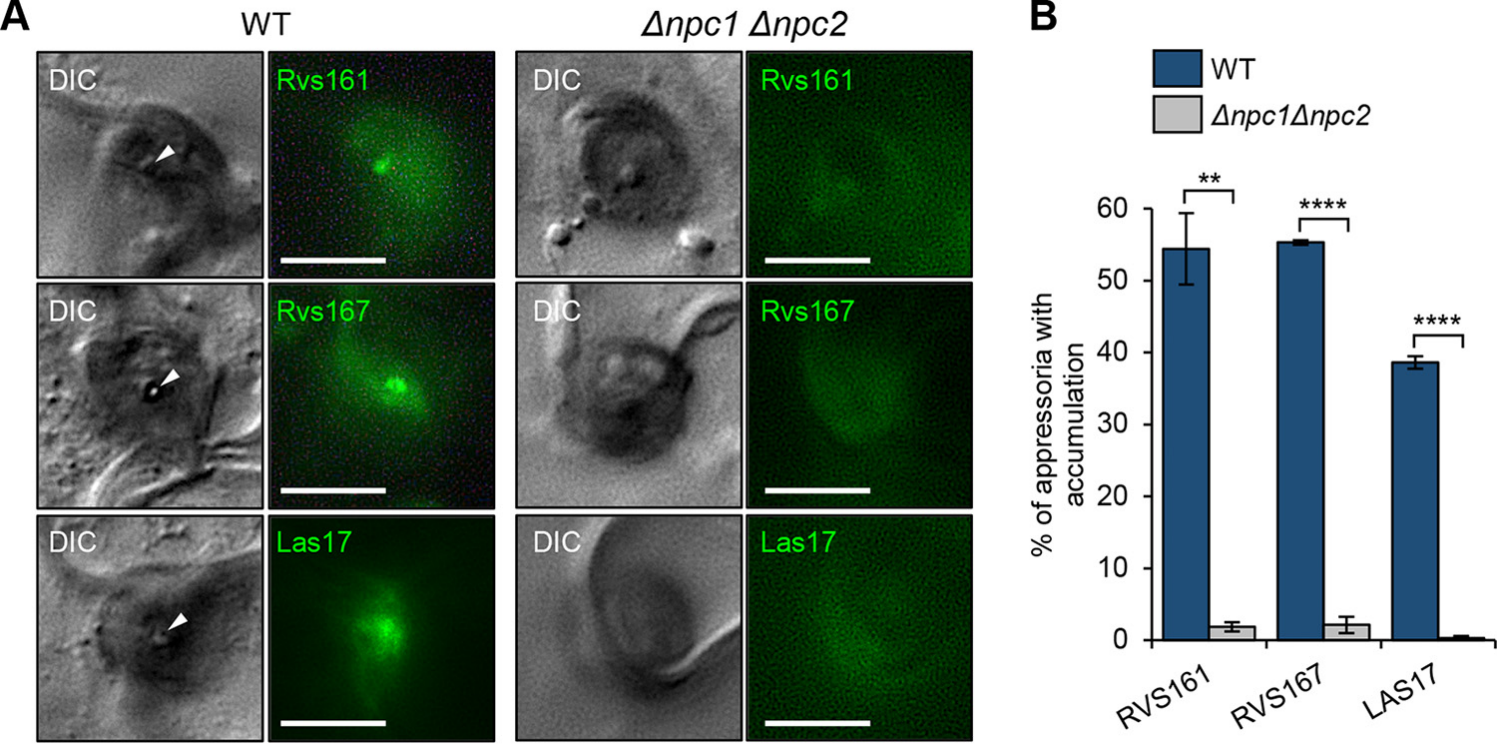
*CoNPC1* and *CoNPC2* are required for membrane curvature necessary for penetration peg emergence. (A) Representative fluorescence micrographs showing the assembly of the I-BAR proteins CoRvs161-GFP and CoRvs167-GFP and the component of the Arp2/3 complex CoLas17-GFP at the appressorial pore. Strains carrying each protein were incubated on a cotyledon for 48 h. Arrowheads, appressorial pore. Scale bars, 5 μm. (B) Mean percentages (± SE; *n *=* *3) of the appressoria show protein assembly at the appressorium pore at 48 hpi. At least 100 appressoria were scored in each replicate. **, *P < *0.01; ****, *P < *0.0001 (Student *t* test).

Based on these findings, we speculated that the deletion of *CoNPC1* and *CoNPC2* affects membrane curvature for protrusion of a penetration peg and appressorial corn formation. The Bin/Amphiphysin/Rvs (BAR) domain protein family is well known to play a role in the formation and sense of membrane curvatures and in attracting the regulators of actin dynamics (37). In M. oryzae, the inverse BAR (I-BAR) protein Rvs167 localized in the appressorium pore and is thought to be involved in membrane protrusion associated with the penetration peg emergence (38). Thus, we evaluated the localization of the I-BAR protein homologs CoRvs161 and CoRvs167 (Fig. 6). CoRvs161-GFP and CoRvs167-GFP both localized at the appressorium pore and that localization was disrupted in a *Δconpc1 Δconpc2* mutant. Furthermore, a homolog of the actin-polymerizing Arp2/3 complex protein CoLas17 (39) also localized at the appressorium pore, and this was impaired in a *Δconpc1 Δconpc2* mutant. This suggests that *CoNPC1* and *CoNPC2* are required for membrane curvature with actin polymerization at the emerging penetration peg and the appressorial cone.

**FIG 7:**
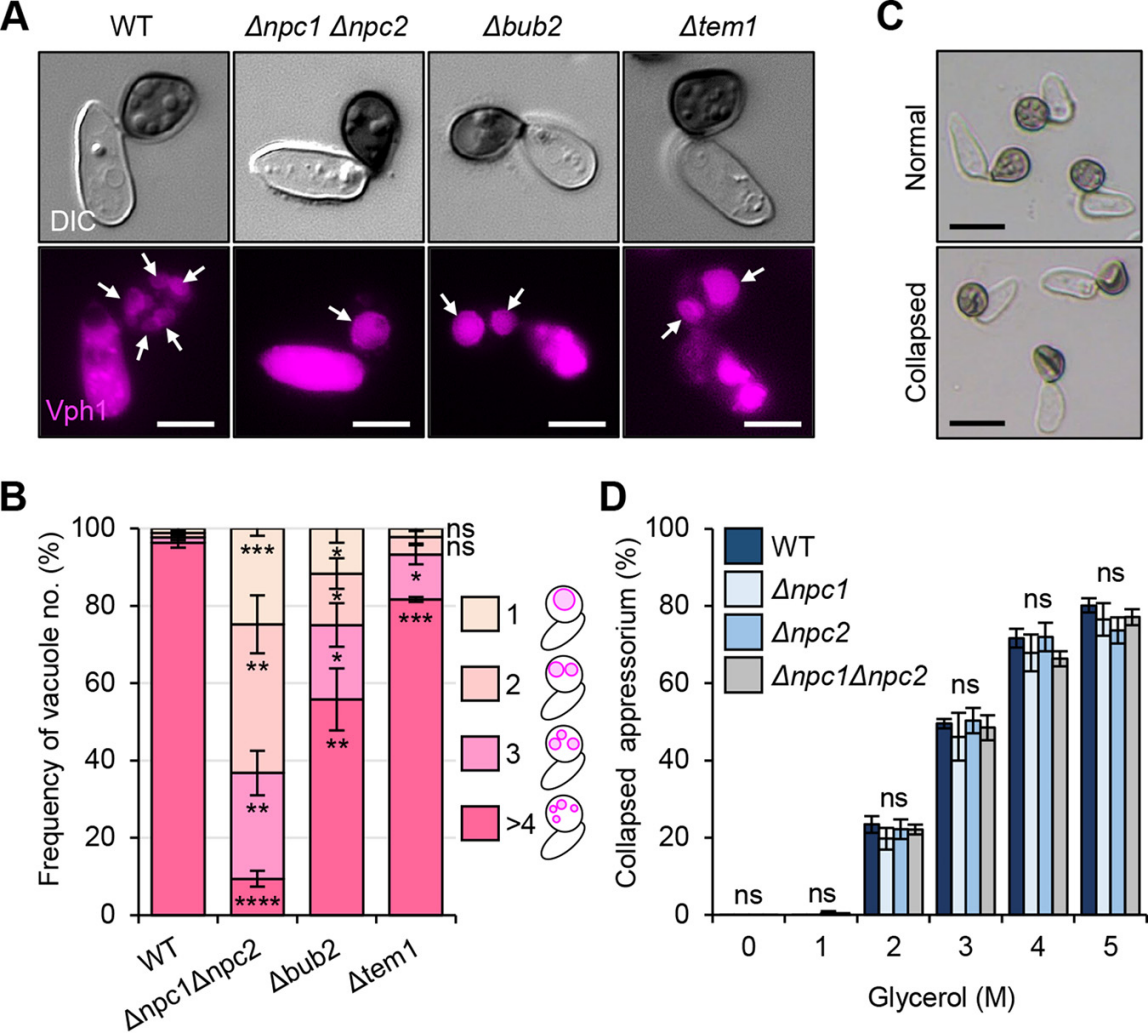
*CoNPC1* and *CoNPC2* are required for vacuolar morphogenesis but not turgor generation in a mature appressorium. (A) Representative fluorescence micrographs of vacuolar morphology in a mature appressorium (24 hpi) of the indicated strains carrying CoVph1-mCherry. White arrows, vacuoles. Scale bars, 5 μm. (B) Frequency of mature appressoria with different numbers of vacuoles. Means ± SE of three independent experiments (>70 appressoria were counted for each replicate) are shown. *, *P < *0.05; **, *P < *0.01; ***, *P < *0.001; ****, *P < *0.0001; ns, not significant (Student *t* test). (C) Representative bright-field micrographs of normal and collapsed appressoria. Scale bars, 10 μm. (D) Cytorrhysis assay to determine appressorial turgor pressure. Mean percentages (± SE, *n *=* *3) of the appressoria that had collapsed. A conidial suspension of strain was incubated for 48 h on glass slides and then exposed to glycerol solutions (0 to 5 M) for 15 min. At least 200 appressoria scored for each replicate. A two-tailed independent *t* test versus the wild type (WT) was applied for all mutant strains (ns, not significant).

We found that the *Δconpc1* and *Δconpc2* mutants form two abnormally enlarged vacuoles in conidia compared to the wild type (Fig. 3E). To check whether similar morphological abnormalities are found in appressoria, we examined the vacuolar morphology in *Δconpc1 Δconpc2* appressoria by labeling of CoVph1-mCherry, a homolog of the integral vacuole membrane protein Vph1, V_0_ subunit of the vacuolar ATPase in S. cerevisiae (40) (Fig. 7A). Vacuoles in the mature appressorium of the *Δconpc1 Δconpc2* mutant were larger in size, spherical, and smaller in number (24 hpi) than in the wild type (Fig. 7A and B), indicating that *CoNPC1* and *CoNPC2* were required for appropriate vacuolar morphogenesis in the appressorium. Intriguingly, the *Δcobub2* and *Δcotem1* mutants showed a decrease in vacuoles in the mature appressorium compared to the wild type (Fig. 7A and B), suggesting that CoBub2-CoBfa1 and CoTem1 cascades are responsible for vacuolar morphogenesis. This implies the functional relevance between CoNpc2 and CoTem1 cascades. We next examined whether the hypertrophied vacuoles in *Δconpc1* and *Δconpc2* mutants affect appressorial turgor. During appressorium maturation, vacuoles take up lipid bodies and enlarge to generate turgor in the appressorium of M. oryzae (41). In the cytorrhysis assay to evaluate the appressorial turgor pressure, mature appressoria were exposed to various concentrations of glycerol, and any collapsed appressoria were counted. The turgor generated by appressoria of the *Δconpc1* and *Δconpc2* mutants was similar to that of the wild type (Fig. 7C and D). Thus, *CoNPC1* and *CoNPC2* does not seem to be involved in producing the internal turgor pressure, and the penetration failure by the appressoria of the two mutants is not due to a reduction in turgor pressure.

## DISCUSSION

**FIG 8:**
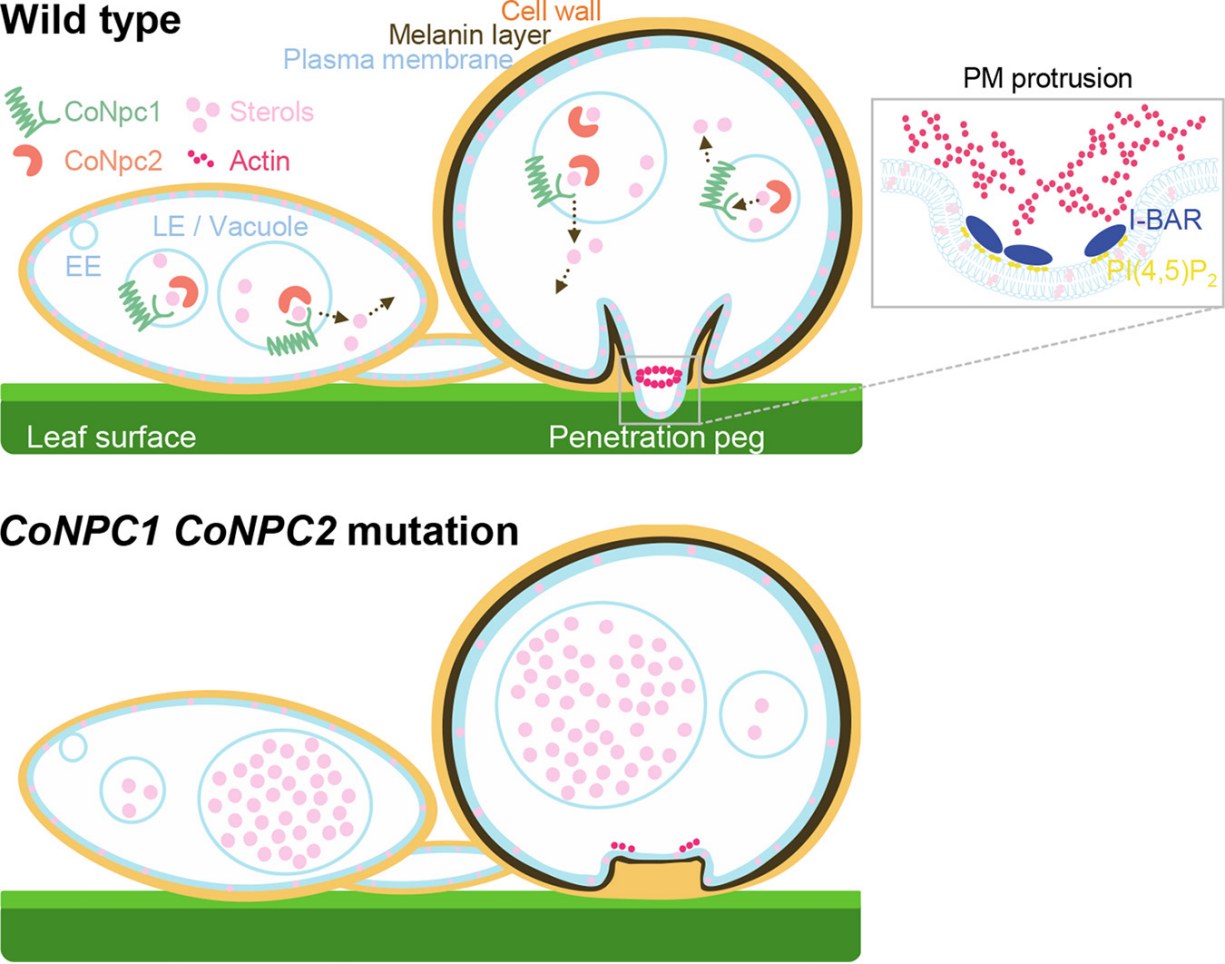
Schematic model of sterol transport by CoNpc1 and CoNpc2 in *C. orbiculare*. CoNpc1 and CoNpc2 are engaged in sterol transport in late endosomes and vacuoles. For infection by the appressorium, sterol transport and proper distribution mediated by CoNpc1 and CoNpc2 are critical for membrane integrity and actin assembly and subsequent penetration peg emergence and appressorial cone formation. A model of plasma membrane protrusion at the site of penetration pore is shown in the boxed area. The I-BAR domain protein with phosphatidylinositol-4,5-bisphosphate [PI(4,5)P_2_] promotes the formation of plasma membrane (PM) protrusions and actin filament assembly. EE, early endosome; LE, late endosome.

Human NPC proteins NPC1 and NPC2 bind with cholesterol and cooperate in sterol transport from late endosomes and lysosomes (29, 42, 43). A mutation in NPC proteins in mammalian cells leads to the accumulation of sterols and other lipids in lysosomes (34). Phylogenetic and homology analyses suggest that NPC proteins are conserved among fungi to humans. Here, CoNpc1-GFP and CoNpc2-mCherry were colocalized in late endosomes and vacuoles. As shown by filipin staining, the *Δconpc1* and *Δconpc2* mutants accumulated sterols in vacuoles but not in the plasma membranes of conidia and appressoria, resulting in enlarged vacuoles of these mutants. When considered together, these results demonstrate that the CoNpc1 and CoNpc2 function in sterol transport from vacuoles for redistribution to plasma membranes in *C. orbiculare* (Fig. 8). Although CoNpc1 has 13 transmembrane domains, similar to human and yeast homologs, we did not observe any distinct vacuolar membrane localization of CoNpc1-GFP. Strains expressing CoNpc1-GFP had fully restored host penetration and virulence, indicating that the CoNpc1-GFP fusion protein is fully functional. Considering that the vacuolar membrane protein CoVph1-mCherry, which has seven transmembrane domains, showed labeling of the vacuolar membrane and lumen, the protein fusion might affect localization of these proteins.

Compared to the preinfection morphogenesis of wild-type *C. orbiculare* (44), the vacuoles of conidia in the *Δconpc1* and *Δconpc2* mutants were enlarged, but germination and appressorial morphogenesis were no different even though penetration of the host plant was impaired and virulence was markedly reduced. The defect in penetration was associated with an aberrant appressorial cone and impaired actin assembly at the appressorial pore and peg formation but not with reduced turgor pressure. These results indicate that sterol transport by NPC proteins is required for appressorium-mediated plant infection of *C. orbiculare*. Many but not all *Colletotrichum* species produce an appressorial cone around the pore (45–47). Although cones are absent from the appressoria of many direct-penetration fungi, including *C. truncatum*, *C. graminicola*, and M. oryzae, some direct penetrators form a similar structure in the appressorium (45, 47). Thus, although the appressorial cone is not necessary for direct penetration, these structures might play an important role in plant penetration by *C. orbiculare*. Because the plasma membrane is closely appressed to the inner cell wall layer and melanin layer of the appressorial cone in *C. orbiculare*, the aberrant cone of the *Δconpc2* mutant could be due to changes in plasma membrane properties that were affected by sterol levels (48, 49). This possibility is in accordance with the loss of filipin staining in the plasma membranes of the *Δconpc1* and *Δconpc2* mutants. Thus, we suggest that proper sterol distribution mediated by CoNpc1 and CoNpc2 contributes to appressorial cone development.

The fungal plasma membrane is known to have several different functions in a wide range of dynamic processes, including environmental sensing, endocytosis, secretion, morphogenesis, cell wall synthesis, and nutrient uptake (15). For these cellular processes, the membrane curvature is essential for the generation of membrane invaginations and protrusions, followed by rapid membrane biogenesis and F-actin polymerization. Curvature can be generated by processes such as local enrichment of specific lipids, protein scaffolding, and binding-protein insertion/interaction (50). One of best-known regulators of membrane curvature is a family of Bin/amphiphysin/Rvs (BAR) domain proteins, which act as connecting links between actin dynamics and membrane rearrangements (37). Members of the classical BAR, N-BAR, and F-BAR proteins subfamily are known to generate membrane invagination through endocytosis, whereas I-BAR proteins are involved in negative membrane curvature, generating a membrane protrusion, which occurs at the appressorial penetration site. In M. oryzae, the proteins Rvs167 I-BAR and Las17, a component of the actin-polymerizing Arp2/3 complex, are localized at the appressorium pore and control the emergence of the appressorial penetration peg (38). In addition, ezrin, radixin, moesin (ERM) domain proteins required for actin-membrane interactions at the cortex of cells, and phosphatidylinositol (PtdIns)-4-kinase and PtdIns-4-phosphate 5-kinase are also localized at the appressorial pore with F-actin. PtdIns-4,5-bisphosphate is required for enhanced phosphorylation of ERM proteins (51), and the I-BAR and BAR domains induce clustering of PtdIns-4,5-bisphosphate upon membrane binding (52, 53). In *C. orbiculare*, penetration peg generation with the appressorial cone would thus be intricately regulated not only by membrane protrusion but also by membrane invagination. We found that the I-BAR proteins CoRvs161-GFP and CoRvs167-GFP and the component of Arp2/3 complex CoLas17-GFP localized to the appressorial pore, and those were not properly organized in the *Δconpc1 Δconpc2* mutant. Furthermore, actin in the mutant was mislocalized at the appressorial pore. Based on these results, we propose that sterol transport mediated by *CoNPC1* and *CoNPC2* would be critical for membrane curvature necessary for penetration peg emergence (Fig. 8). On the other hand, a deficiency in *CoNPC1* and *CoNPC2* did not affect turgor pressure of the appressorium. While *CoNPC1* and *CoNPC2* are necessary for appressorial penetration, sterol transport by NPC proteins is not involved in appressorial turgor generation.

While *CoNPC1* and *CoNPC2* are necessary to appressorial penetration, they were not responsible for hyphal growth inside the plant tissues and the media. This is consistent with the yeast NPC proteins are required for sterol transport, especially under starvation conditions (28). Because the ergosterol biosynthesis needs a considerable amount of energy (54), when appressoria form on the nutrient-free leaf surface, proper sterol distribution of *C. orbiculare* may be maintained by the recycling from vacuole via NPC proteins. On the other hand, hyphal sterol homeostasis may rely on endogenous synthesis and uptake from exogenous sources.

We previously reported that GTPase CoTem1 regulates proper G_1_/S progression via two-component GAP CoBub2-CoBfa1 in *C. orbiculare* (3). In this study, we showed that CoNpc2, the physical interaction factor of CoTem1, is not related to G_1_/S progression and nuclear autophagy. In addition, CoBub2-CoBfa1 and CoTem1 are not involved in sterol transport. There are no reports that Npc2 homologs are involved in G_1_/S progression or that Tem1 homologs are involved in sterol transport. Based on their physical interaction, CoTem1 and CoNpc2 would be expected to be localized in the same cellular compartment; however, CoTem1 is localized at spindle pole bodies (SPBs) in the nucleus (3), and we showed here that CoNpc2 is localized in the late endosomes and vacuoles. We thus suspected that CoNpc2 yielded a false-positive result in the Y2H assay screening for a physical interaction with CoTem1. To test this possibility, we checked the specificity of the CoTem1-CoNpc2 interaction using the point mutation in the presumable effector binding site. A previous study reported that a mutation in the effector binding site in S. pombe Spg1, a homolog of CoTem1, results in a marked loss of specific interactions with Cdc7, as demonstrated by analysis of the phenotypes of the point mutant and Y2H (9). The point mutation in presumable effector binding site T146A in CoTem_196-302_ resulted in a marked loss of interaction with CoNpc2, indicating a specific interaction between CoNpc2 and CoTem1 at the effector binding site. Consistent with the Y2H test, physical interaction between CoTem1 and CoNpc2 was confirmed by Co-IP assay. In addition, the deletions of *CoNPC1*, *CoNPC2*, *CoBUB2*, and *CoTEM1* genes share characteristic defects in appressorial vacuolar morphogenesis. Based on these results, we speculate an intricate relationship between CoTem1 and CoNpc2 that is mediated by interorganelle communication. Previous reports in budding yeast show that the nucleus-vacuole junctions (NVJs) connect the vacuole with the perinuclear ER and thus the nuclear envelope (55). NVJ formation depends on the nuclear membrane protein Nvj1 and the vacuolar membrane protein Vac8. NVJs allow the recruitment of proteins involved in lipid metabolism and transport and serve as a platform for lipid droplet biogenesis upon glucose restriction (56). NVJ components include the sterol-transfer protein Lam6 and the oxysterol-binding protein Osh1, suggesting its contribution to sterol homeostasis (56). Although NPC proteins were not identified as the NVJ-associated proteins, interactions between NPC2, NPC1, and the ER integral membrane protein ORP5 suggested a potential mechanism for sterol transfer between endosome lumen and the ER in mammals (57). In addition, budding yeast SUN protein family Mps3 localizes at SPBs and affects lipid homeostasis, suggesting its role for modulating the nuclear envelope composition to facilitate insertion of SPBs in the nuclear membrane (58). Though it remains unclear why CoNpc2 interacts with CoTem1, our findings suggest that intracellular sterol transport mediated by CoNpc2 and CoNpc1 performs a critical role in the CoBub2-CoBfa1 and CoTem1 cascade for appressorium penetration.

## MATERIALS AND METHODS

### Fungal and bacterial strains and culture conditions.

*C. orbiculare* strain 104-T (MAFF240422) was used as the wild-type strain. All strains used in this study are listed in Table S2A in the supplemental material and were cultured at 24°C on 3.9% potato dextrose agar (PDA; Nissui, Japan). For genetic manipulation, Escherichia coli DH5α-competent cells were maintained at 37°C on Luria-Bertani (LB) agar. For fungal transformation, Agrobacterium tumefaciens C58C1 was maintained at 28°C on LB agar, and *C. orbiculare* was transformed as previously described (3, 36), with the following slight modifications. Geneticin-resistant transformants were selected on PDA containing 50 μg/mL G418 sulfate (Wako Chemicals, Japan) and 25 μg/mL meropenem hydrate (Sumitomo Dainippon Pharma, Japan).

10.1128/mbio.02236-22.10 TABLE S2 Fungal strains and primers used in this study. Fungal strains (A) and primers (B), respectively, are listed. Download Table S2, DOCX file, 0.06 MB. Copyright © 2022 Kodama et al. 2022 Kodama et al. https://creativecommons.org/licenses/by/4.0/ This content is distributed under the terms of the Creative Commons Attribution 4.0 International license.

### Strain construction.

For generating the *CoNPC2* disruption mutant, the 1.0-kb upstream and downstream flanking sequences and a 1.4-kb hygromycin-resistance gene cassette were amplified and introduced into linearized binary vector pBIG4MRBrev using the In-Fusion HD Cloning kit (TaKaRa Bio USA, Inc., USA). The same procedure was applied to generate the *CPI1* to *CPI10* disruption mutants. For generating the *CoNPC1* disruption mutant, the 1.0-kb upstream and downstream flanking sequences and a 1.0-kb neomycin-resistance gene cassette were amplified and introduced into linearized pPZP-PvuII. Gene deletion of mutants was confirmed by genomic PCR analysis using the two primer pairs listed in Table S2B. For constructing CoNpc1-GFP and CoNpc2-mCherry gene fusions, *CoNPC1* and *CoNPC2* complementation vectors were first constructed. *CoNPC1* and *CoNPC2* gene fragments containing the 1.0-kb upstream and downstream flanking sequences were inserted into the linearized pBIG4MRSrev and pBIG4MRBrev, respectively. Then, the green fluorescent protein (GFP) and the mCherry fragments were inserted at the C-terminal end of *CoNPC1* and *CoNCP2* in these complementation vectors. The same procedure was applied to generate CoRab7-GFP, CoVph1-mCherry, CoRvs161-GFP, CoRvs167-GFP and CoLas17-GFP gene fusions. To construct the CoNpc2-MYC overexpression strains, a 4.0-kb *CoNPC2-3MYC* fragment containing its 1.1-kb downstream flanking sequence was amplified and fused to linearized pCAMSUR-TEF containing the translation elongation factor promoter of *Aureobasidium pullulans*. The same procedure was applied to generate CoNpc1-MYC and CoTem1-HA gene fusions. The primers and plasmids used in this study are listed in Table S2B.

### Pathogenicity assay.

For pathogenicity testing, six 10-μL drops of a conidial suspension (1 × 10^5^ conidia/mL in distilled water) of *C. orbiculare* were placed on detached cucumber leaves (*Cucumis sativus* L. “Suyo”) in a humid box and incubated at 24°C for 5 days. To test the invasive growth ability of the fungus, 1-μL drops of a conidial suspension (5 × 10^5^/mL in distilled water) were dropped onto wound sites prepared by scratching the leaf surface with a sterile toothpick, and then the leaves were incubated as described. Cucumber plants were maintained in a growth chamber (16 h light/8 h dark, 24°C).

### Microscopy.

Cucumber cotyledons were inoculated in a humid box with 5 × 10^5^ conidia/mL in distilled water, incubated at 24°C for 72 h, and then stained with 0.1% (wt/vol) lactophenol aniline blue solution, as described previously, in order to observe any penetration hyphae (59, 60). To induce papillae on non-host onion epidermis, a conidial suspension (1 × 10^5^ conidia/mL in distilled water) was dropped onto the abaxial surface of split pieces of onion bulb in a humid box. After 24 to 72 h at 24°C, the epidermis was peeled off and observed. A cytorrhysis assay was used as previously described to determine appressorial turgor (61).

To observe the conidial and appressorial morphology of *C. orbiculare*, a conidial suspension (5 × 10^5^ conidia/mL) was placed on a multiwell glass slide (Matsunami Glass, Japan) and incubated in a humid box at 24°C in the dark for 10 min to 24 h. To stain cellular membranes and vacuolar lumen, fungal cells were incubated with 5 μg/mL FM 4-64, 10 μM CellTracker Green CMFDA, and 10 μM CellTracker Blue CMAC (all from Thermo Fisher Scientific, USA) in distilled water for 10 min and then rinsed with distilled water. For staining sterol, cells were stained with 0.5 mg/mL filipin III (Sigma-Aldrich, USA) immediately before microscopy. For nuclear staining, samples were fixed in 4% (wt/vol) paraformaldehyde in 100 mM phosphate-buffered saline (PBS) containing 0.2% Triton X-100. Fixed cells were then washed twice with 100 mM PBS, and 1 μL of 1-μg/mL DAPI (Sigma-Aldrich) was added directly to the cells on slides. A Zeiss Axio Imager M2 upright microscope (Carl Zeiss, Germany) equipped with a Plan Apochromat 100× oil immersion lens and an Axio Cam MRm digital camera was used to acquire fluorescence images. Excitation/barrier filters were set at 470 nm/509 nm for GFP and 595 nm/620 nm for RFP. Images were acquired using AxioVision 4.8 software (Carl Zeiss, Germany). Fluorescence intensity profiles were created using ImageJ software (http://rsb.info.nih.gov/ij/). Area measurements of vacuolar fluorescent staining were conducted using an All-in-One BZ-X800 (Keyence) fluorescence microscope equipped with Plan Fluorite 40× LD PH phase lenses (Keyence). An excitation wavelength of 470/40 nm, an emission wavelength 525/50 nm, and a dichroic mirror wavelength of 495 nm were used for CMFDA. Fluorescent microscopic images were acquired using BZ-H4A software (Keyence) and analyzed using BZ-H4M software (Keyence). Bright-field microscopy was performed using a Nikon Eclipse E600 microscope equipped with a 40× water immersion lens (Nikon, Japan) and a DP74 digital camera system (Olympus, Japan). For papilla observations, an excitation wavelength of 365/10 nm, a dichroic mirror wavelength of 400 nm, and a barrier filter wavelength of 400 nm were used.

### Yeast two-hybrid interaction assays.

The Gold Yeast Two-Hybrid System (TaKaRa Bio USA, Inc., USA) was used. In a library-scale Y2H system, the prey strains carried a *C. orbiculare* cDNA library from vegetative mycelia and conidia, and the bait strains carried cDNA of CoTem_196-302_. In a one-to-one-scale Y2H system, Cpi1 to Cpi10 were expressed in fusion with the Gal4 DNA-binding domain (BD) protein, and their interactions with CoTem1_96-302_ fused with the Gal4 activation domain (AD) were tested in the Y2H assay as described previously (62).

### Co-IP assays.

The *C. orbiculare* strains used for Co-IP assays *in vivo* were CoTem1-HA, CoNpc1-MYC, CoNpc2-MYC, CoTem1-HA/CoNpc1-MYC, and CoTem1-HA/CoNpc2-MYC (see Table S2A). Total protein was extracted from vegetative mycelia using radioimmunoprecipitation assay (RIPA) buffer (50 mM Tris-HCl [pH 8.0], 150 mM NaCl, 1% Nonidet P-40, 0.5% sodium deoxycholate, and a protease inhibitor cocktail tablet), followed by incubation with Pierce Anti-HA magnetic beads (Thermo Fisher Scientific, USA) at 24°C for 1 h. After a washing step with RIPA buffer, the eluted proteins were separated by SDS-PAGE using a NuPAGE 4 to 12% Bis-Tris gel (Thermo Fisher Scientific) and transferred to a polyvinylidene difluoride membrane (Thermo Fisher Scientific). Western blotting with anti-HA and anti-MYC antibody was performed as previously described (62) with the following modifications. The primary antibodies were diluted 1:1,000 (anti-HA, H6908; Sigma-Aldrich) in Tris-buffered saline with 0.1% Tween (TBST) or 1:1,000 (anti-MYC, SAB4301136; Sigma-Aldrich) in 5% skim milk TBST and incubated at 24°C for 2 h. Membranes were imaged by using a ChemiDoc Touch MP Imaging System (Bio-Rad, USA).

### Phylogenetic analyses.

Based on the amino acid sequences of NPC1 and NPC2 homologs obtained from the National Center for Biotechnology Information (www.ncbi.nlm.nih.gov), alignments were created using ClustalW (63). Phylogenetic dendrograms were constructed using MEGA X (64), with the minimum evolution algorithms using 1,000 bootstrap replications.

### Transmission electron microscopy.

To observe the penetration peg and appressorial cone on cotyledons, samples were fixed overnight in 4% (vol/vol) paraformaldehyde and 2% (vol/vol) glutaraldehyde in 0.05 M cacodylate buffer (pH 7.4) at 4°C. After fixation, samples were washed with 0.05 M cacodylate buffer and postfixed with 2% (wt/vol) OsO_4_ in 0.05 M cacodylate buffer at 4°C. The samples were dehydrated in a graded ethanol series (50 to 100%) at room temperature and then in 100% propylene oxide (PO). Samples were then soaked in a 70:30 mixture of PO and resin (Quetol-651; Nisshin EM Co., Japan) for 1 h, and then the PO was allowed to volatilize overnight. Samples were transferred to fresh 100% resin and placed in an oven at 60°C for 48 h. Ultrathin sections (80 nm) were cut from the polymerized resin blocks using an ultramicrotome (Ultracut UCT; Leica, Germany), mounted on Formvar-coated copper grids (F-150 Cu; Nisshin EM Co., Japan), and stained with 2% uranyl acetate and lead stain solution (Sigma-Aldrich). Sections were observed with a JEM-1400Plus transmission electron microscope (JEOL Ltd., Japan) and an acceleration voltage of 100 kV. Digital images were taken with a CCD camera EM-14830RUBY2 (JEOL Ltd.). To observe conidial vacuoles, the samples sandwiched with the copper disks were quick-frozen in liquid propane (–175°C) and then freeze-substituted with 2% OsO_4_ in acetone and 2% distilled water at −80°C for 48 h. Samples were transferred to −20°C and then to 4°C. After 3 h, the samples were dehydrated in anhydrous acetone and 100% ethanol at room temperature. The samples were next subjected to the same protocol used for the dehydrated appressorium samples above.

### Data availability.

All data presented here are available from the corresponding author upon reasonable request. Sequence data in this study can be found in the GenBank/EMBL database under the indicated accession numbers: *C. orbiculare* CoNpc1 (TDZ25151), CoNpc2 (TDZ22676), CoRab7 (TDZ22376), CoVph1 (TDZ25208), CoRvs161 (TDZ16133), CoRvs167 (TDZ26074), and CoLas17 (TDZ21267).
