# Supplementary figures and images for: Niemann-Pick Type C Proteins Are Required for Sterol Transport and Appressorium-Mediated Plant Penetration of Colletotrichum orbiculare
Source: mBio. 2022 Sep 26;13(5):e02236-22. doi: 10.1128/mbio.02236-22 (PMC9600679; doi:10.1128/mbio.02236-22)

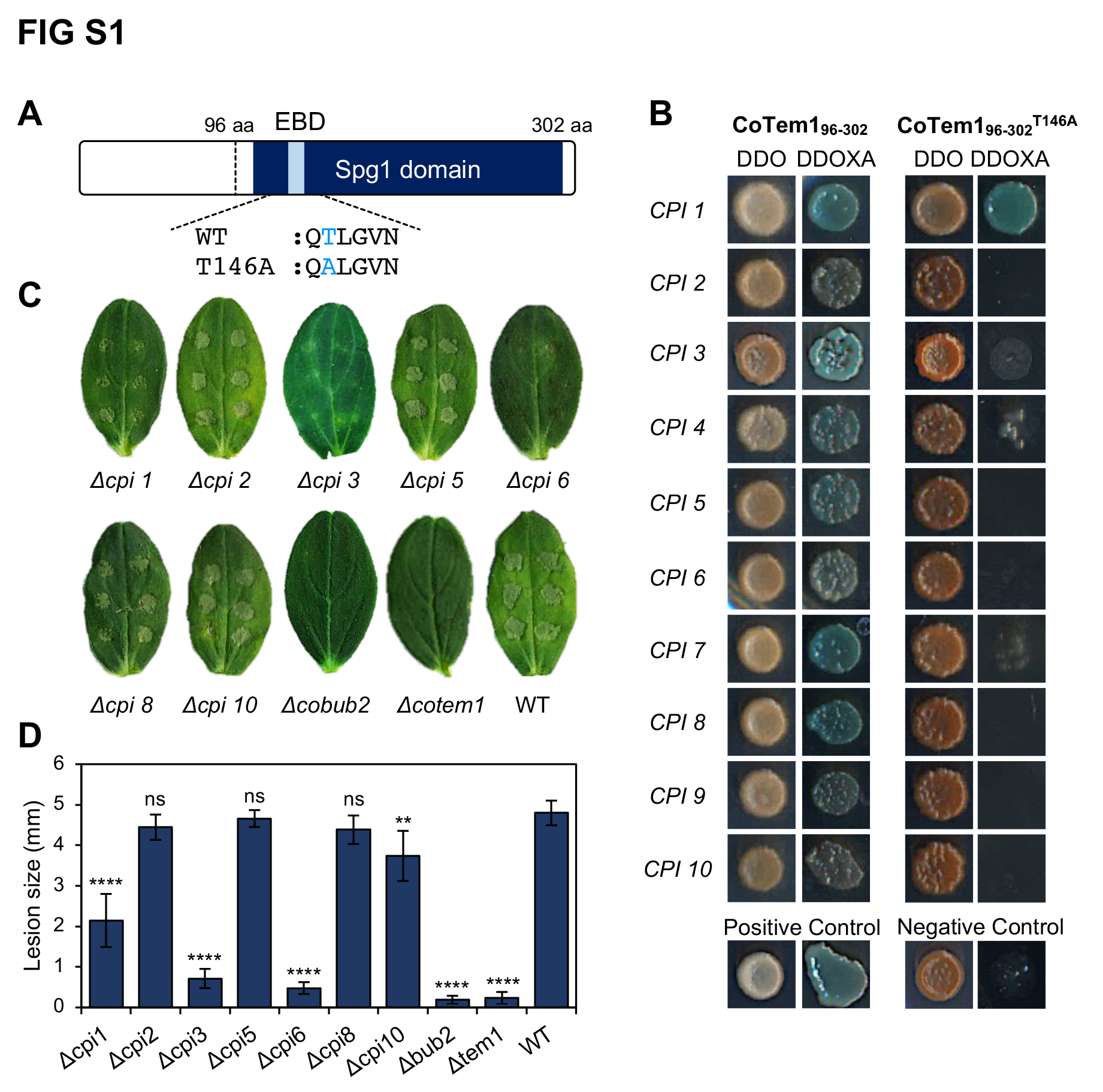

Supplement: FIG S1 [file mbio.02236-22-s0001.tif]

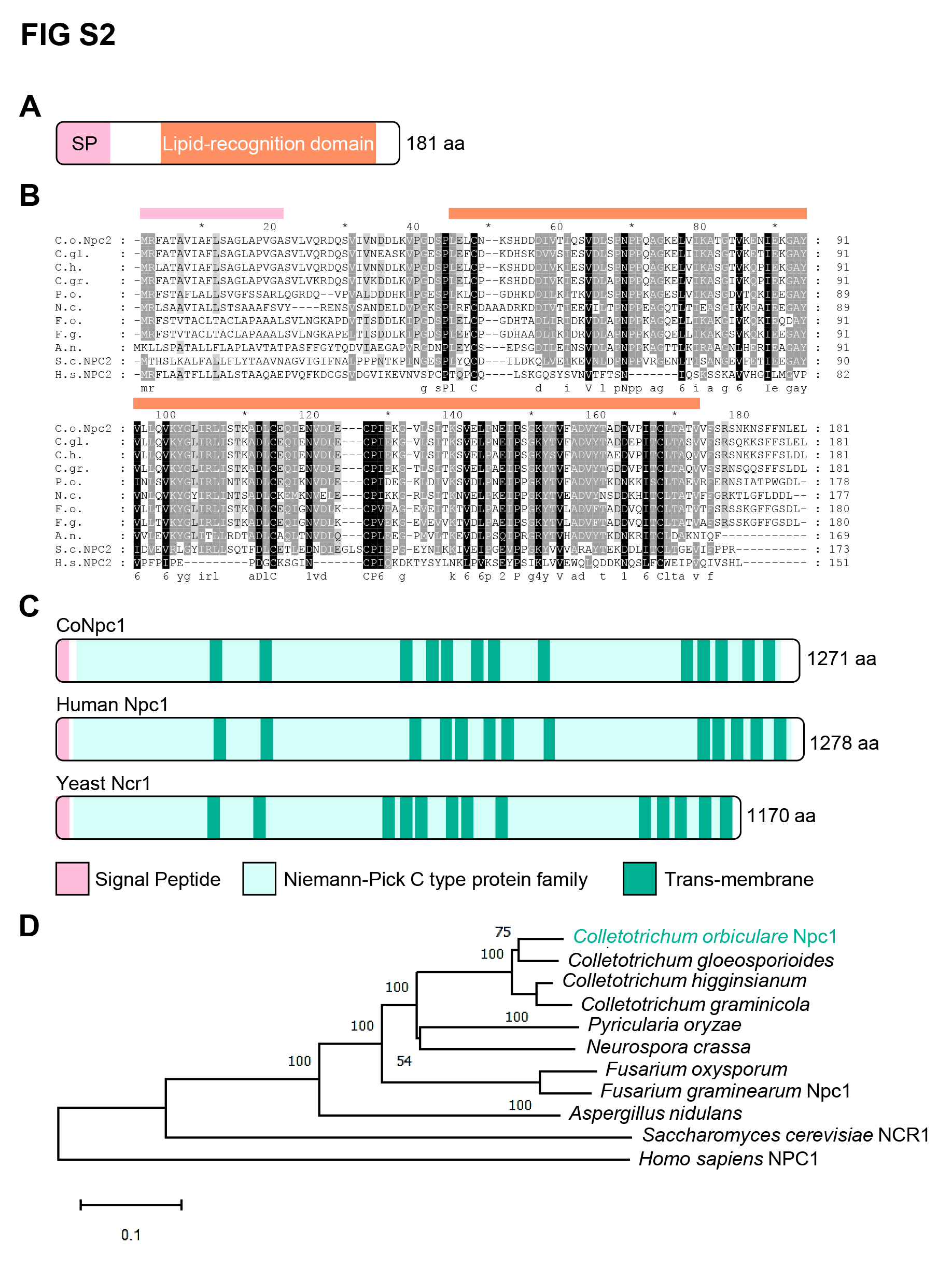

Supplement: FIG S2 [file mbio.02236-22-s0002.tif]

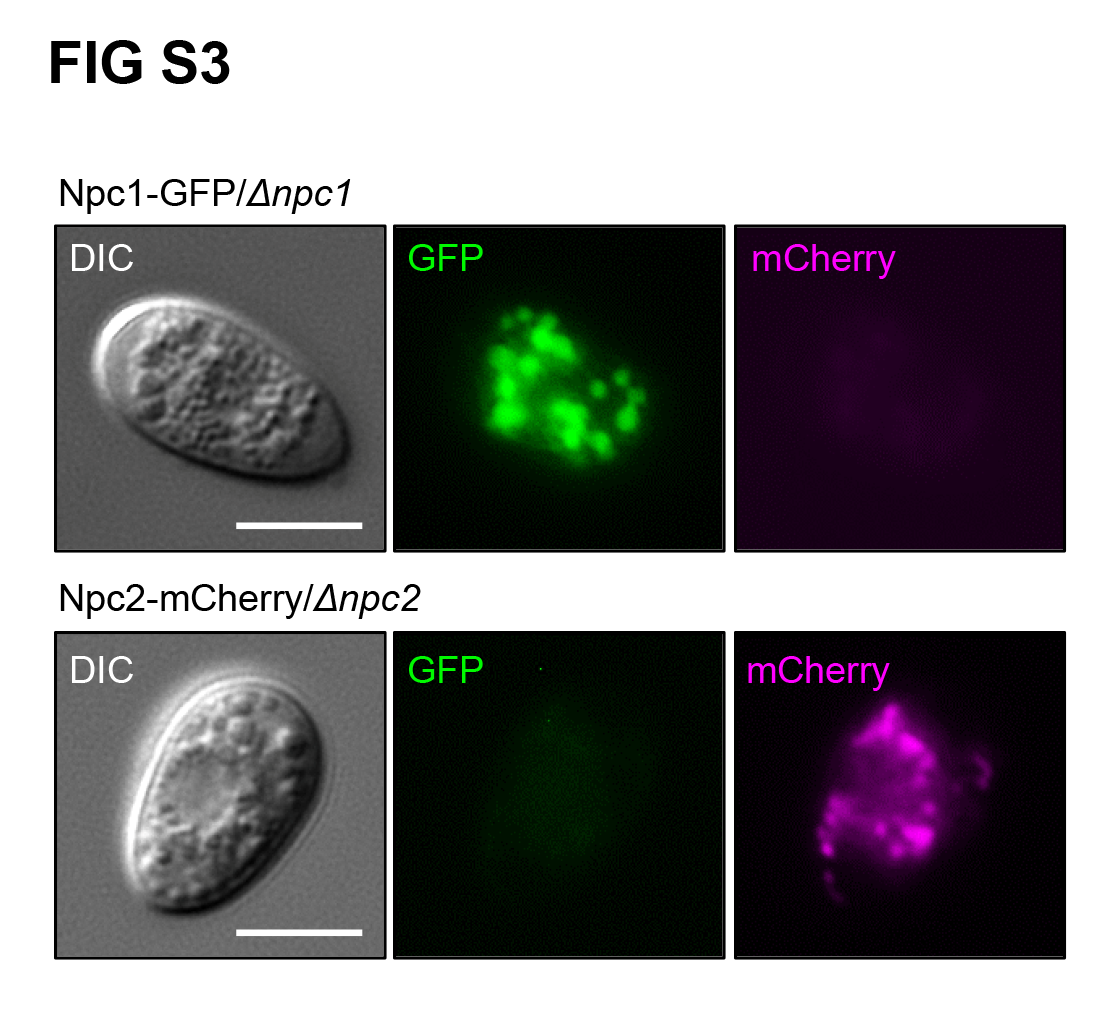

Supplement: FIG S3 [file mbio.02236-22-s0003.tif]

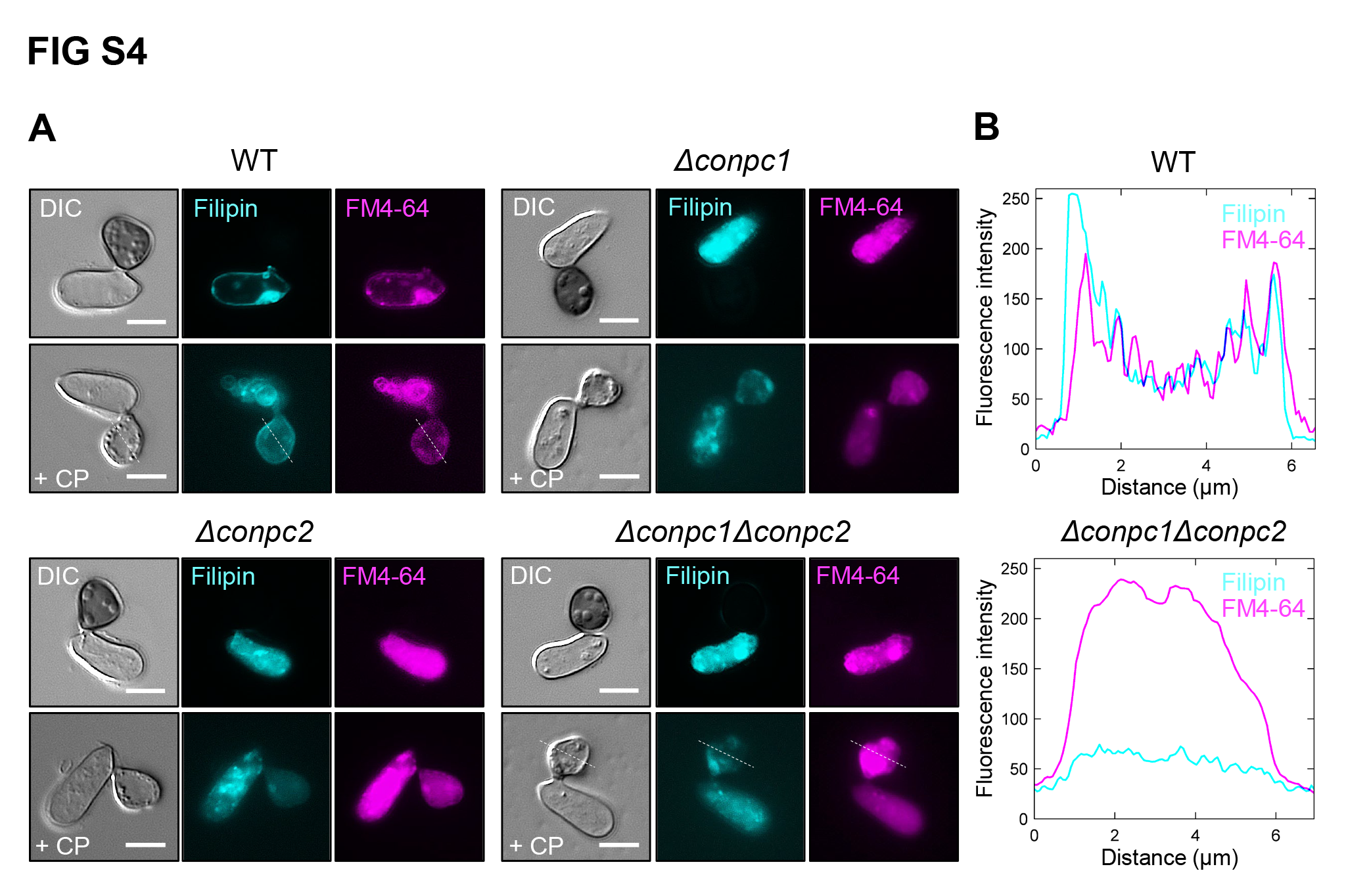

Supplement: FIG S4 [file mbio.02236-22-s0004.tif]

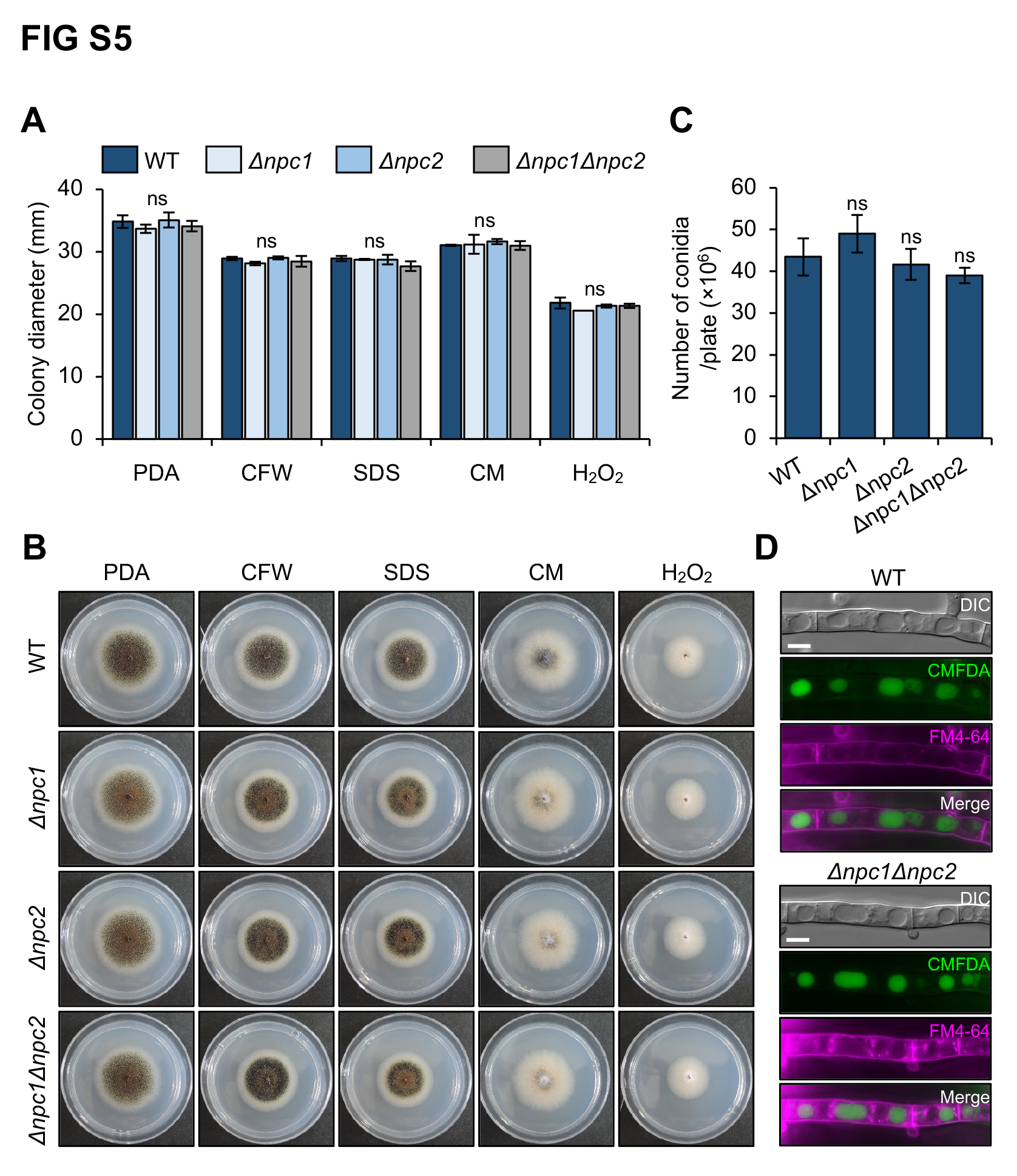

Supplement: FIG S5 [file mbio.02236-22-s0005.tif]

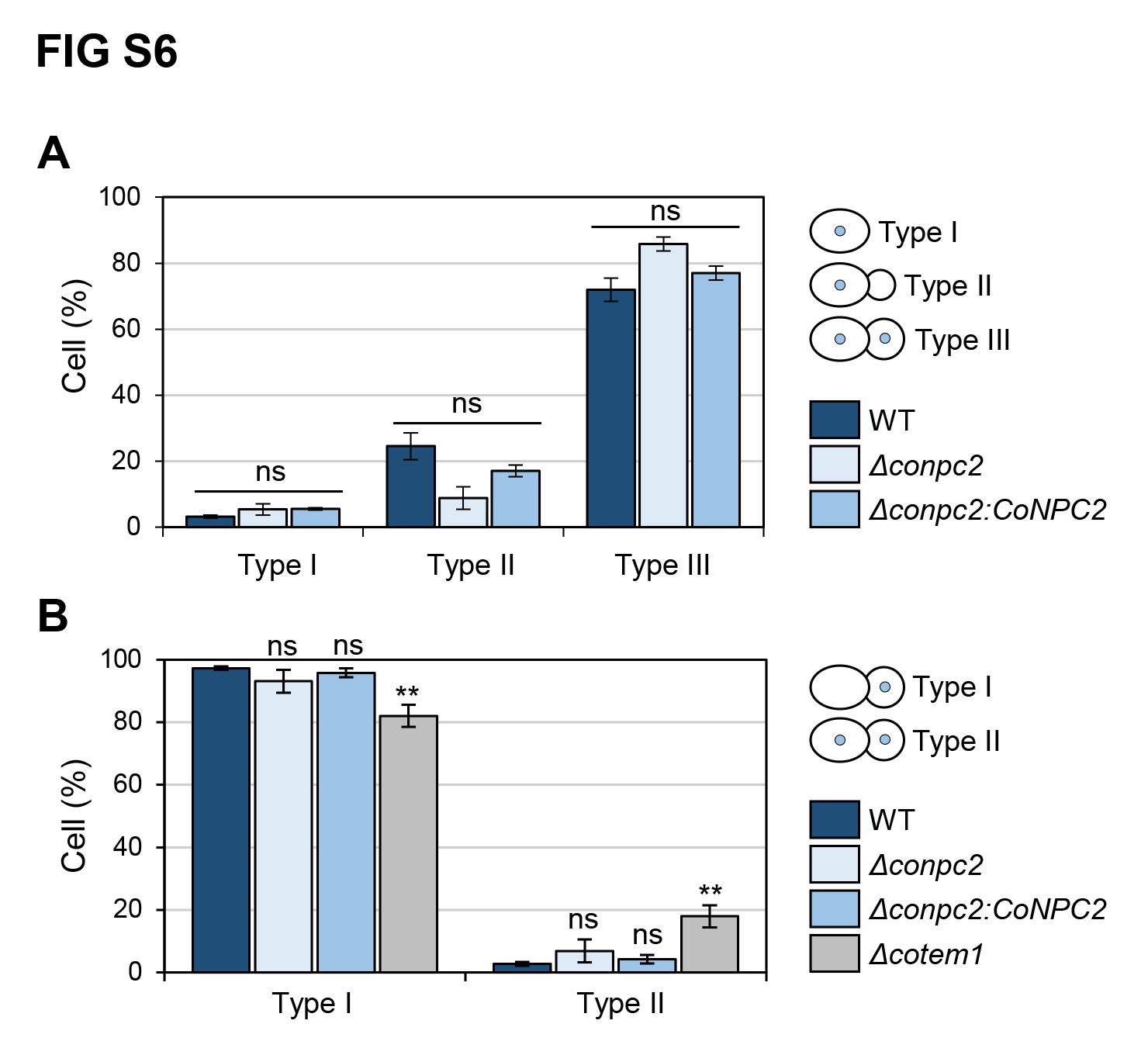

Supplement: FIG S6 [file mbio.02236-22-s0006.tif]

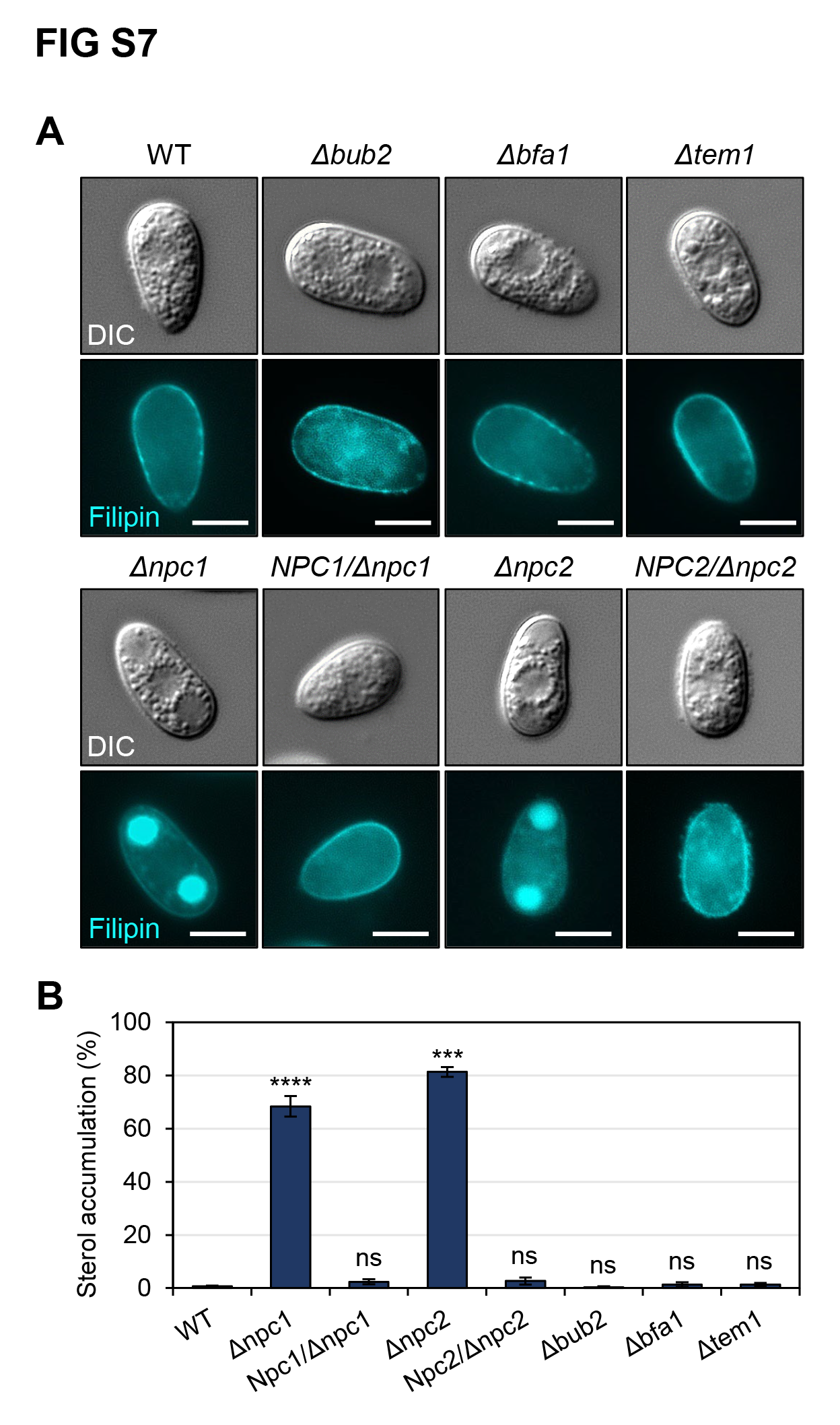

Supplement: FIG S7 [file mbio.02236-22-s0007.tif]

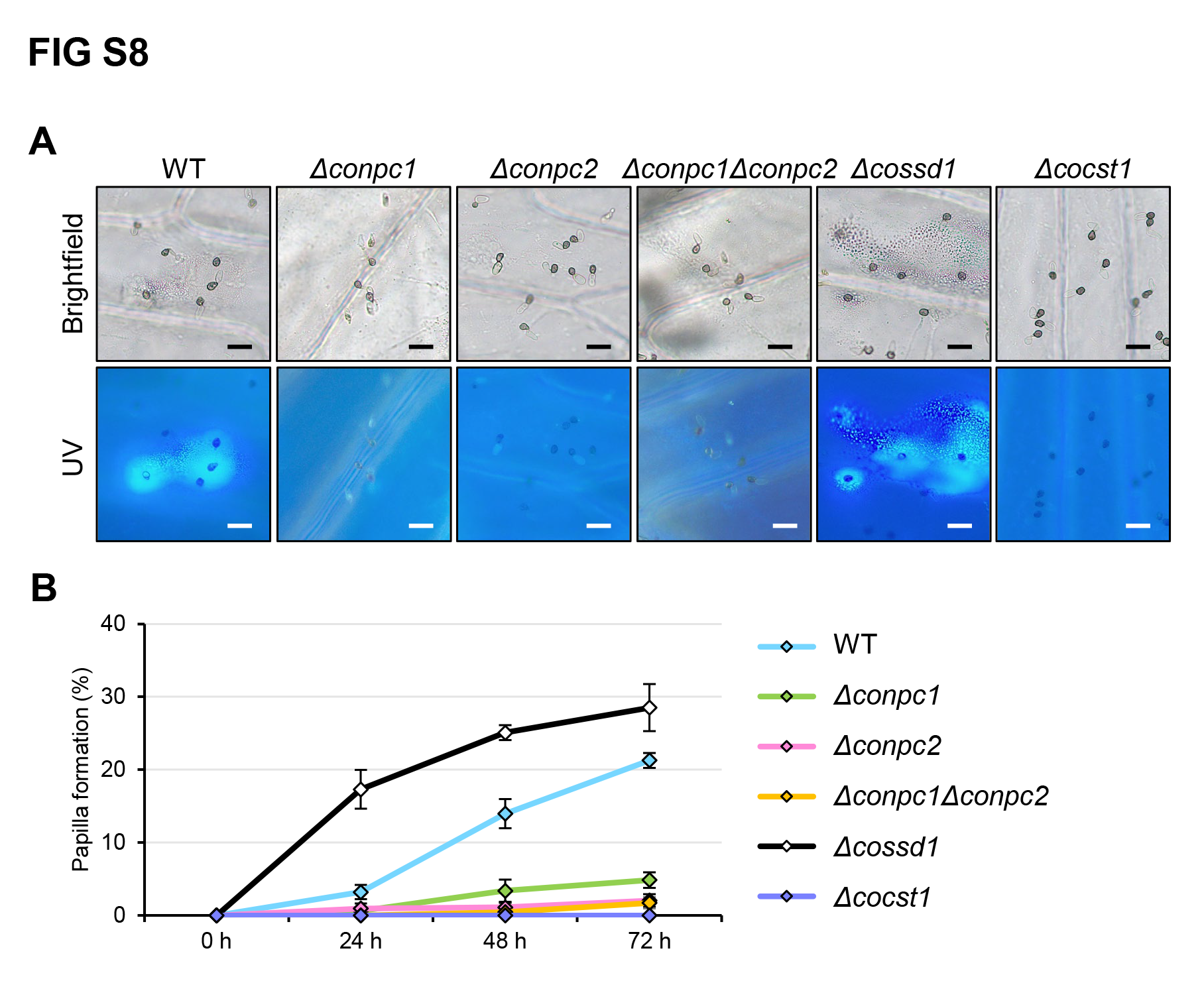

Supplement: FIG S8 [file mbio.02236-22-s0008.tif]
