# Supplementary material for: Niemann-Pick Type C Proteins Are Required for Sterol Transport and Appressorium-Mediated Plant Penetration of Colletotrichum orbiculare
Source: mBio. 2022 Sep 26;13(5):e02236-22. doi: 10.1128/mbio.02236-22 (PMC9600679; doi:10.1128/mbio.02236-22)
Supplement: TABLE S1 [file mbio.02236-22-s0009.docx]

**TABLE S1** List of physical interaction factors with CoTem196-302 in yeast two-hybrid assay.

| Gene Name | Accession no. | Gene description |
| --- | --- | --- |
| *CPI 1* | TDZ25266 | elongation factor tu gtp binding domain protein |
| *CPI 2* | ENH88460 | conidiation-specific protein-8 |
| *CPI 3* | TDZ21250 | hsp70 family protein |
| *CPI 4* | TDZ24667 | nuclear migration protein |
| *CPI 5* | TDZ26710 | gpi anchored serine-threonine rich protein |
| *CPI 6*  (*CoNPC2*) | TDZ22676 | phosphatidylglycerol phosphatidylinositol transfer protein |
| *CPI 7* | TDZ14493 | survival factor 1 |
| *CPI 8* | TDZ14366 | ste12 interacting protein |
| *CPI 9* | TDZ22446 | actin lateral binding protein |
| *CPI 10* | TDZ18104 | helix-loop-helix dna-binding domain-containing protein |
