# Supplementary material for: Niemann-Pick Type C Proteins Are Required for Sterol Transport and Appressorium-Mediated Plant Penetration of Colletotrichum orbiculare
Source: mBio. 2022 Sep 26;13(5):e02236-22. doi: 10.1128/mbio.02236-22 (PMC9600679; doi:10.1128/mbio.02236-22)
Supplement: TABLE S2 [file mbio.02236-22-s0010.docx]

**TABLE S2A** Fungal strains used in this study.

*Colletotrichum orbiculare* strains

| Strain name | Genotype description | Reference |
| --- | --- | --- |
| 104-T (MAFF240422) | Wild type | Ishida and Akai, 1969 |
| Δcpi1 | Wild type / Δcpi1 | This study |
| Δcpi2 | Wild type / Δcpi2 | This study |
| Δcpi3 | Wild type / Δcpi3 | This study |
| Δcpi5 | Wild type / Δcpi5 | This study |
| Δcpi6 (= Δconpc2) | Wild type / Δcpi6 (= Δconpc2) | This study |
| Δcpi8 | Wild type / Δcpi8 | This study |
| Δcpi10 | Wild type / Δcpi10 | This study |
| Δconpc1 | Wild type / Δconpc1 | This study |
| Δconpc1 Δconpc2 | Wild type / Δconpc1 / Δconpc2 | This study |
| CoNPC1 / Δconpc1 | Wild type / Δconpc1 / CoNPC1 | This study |
| CoNPC2 / Δconpc2 | Wild type / Δconpc2 / CoNPC2 | This study |
| CoNpc1-GFP / Δconpc1 | Wild type / Δconpc1 / CoNpc1-GFP | This study |
| CoNpc2-mCherry / Δconpc2 | Wild type / Δconpc2 / CoNpc2-mCherry | This study |
| CoNpc1-GFP / CoNpc2-mCherry | Wild type / CoNpc1-GFP / CoNpc2-mCherry | This study |
| CoRab7-GFP / CoNpc2-mCherry / Δconpc2 | Wild type / Δconpc2 / CoNpc2-mCherry / CoRab7-GFP | This study |
| Lifeact-RFP | Wild type / Lifeact-RFP | Fukada and Kubo, 2015 |
| Lifeact-RFP / Δconpc1 | Wild type / Δconpc1 / Lifeact-RFP | This study |
| Lifeact-RFP / Δconpc2 | Wild type / Δconpc2 / Lifeact-RFP | This study |
| Lifeact-RFP / Δconpc1 Δconpc2 | Wild type / Δconpc1 / Δconpc2 / Lifeact-RFP | This study |
| CoVph1-mCherry | Wild type / CoVph1-mCherry | This study |
| CoVph1-mCherry / Δconpc1 Δconpc2 | Wild type / CoVph1-mCherry / Δconpc1 / Δconpc2 | This study |
| CoRvs161-GFP | Wild type / CoRvs161-GFP | This study |
| CoRvs161-GFP / Δconpc1 Δconpc2 | Wild type / CoRvs161-GFP / Δconpc1 / Δconpc2 | This study |
| CoRvs167-GFP | Wild type / CoRvs167-GFP | This study |
| CoRvs167-GFP / Δconpc1 Δconpc2 | Wild type / CoRvs167-GFP / Δconpc1 / Δconpc2 | This study |
| CoLas17-GFP | Wild type / CoLas17-GFP | This study |
| CoLas17-GFP / Δconpc1 Δconpc2 | Wild type / CoLas17-GFP / Δconpc1 / Δconpc2 | This study |
| CoTem1-HA | Wild type / CoTem1-3HA | This study |
| CoNpc1-MYC | Wild type / CoNpc1-3MYC | This study |
| CoNpc2-MYC | Wild type / CoNpc2-3MYC | This study |
| CoTem1-HA / CoNpc1-MYC | Wild type / CoTem1-3HA / CoNpc1-3MYC | This study |
| CoTem1-HA / CoNpc2-MYC | Wild type / CoTem1-3HA / CoNpc2-3MYC | This study |
| Δcobub2 | Wild type / Δcobub2 | Fukada and Kubo, 2015 |
| Δcobfa1 | Wild type / Δcobfa1 | Fukada and Kubo, 2015 |
| Δcotem1 | Wild type / Δcotem1 | Fukada and Kubo, 2015 |
| Δcocst1 | Wild type / Δcocst1 | Tsuji et al., 2003 |
| Δcossd1 | Wild type / Δcossd1 | Tanaka et al., 2007 |

*Saccharomyces cerevisiae* strains

| Strain name | Genotype description | Reference |
| --- | --- | --- |
| Y2H Gold | MATa, trp1-901, leu2-3, 112, ura3-52, his3-200 | Clontech |
| Y187 | MATα, ura3-52, his3-200, ade2-101, trp1-901, leu2-3, 112, met- | Clontech |
| Y2H Gold/pGBKT7 | MATa, trp1-901, leu2-3, 112, ura3-52, his3-200 pGBKT7 | This study |
| Y2H Gold/pGBKT7-CPI1 | MATa, trp1-901, leu2-3, 112, ura3-52, his3-200 pGBKT7-CPI1 | This study |
| Y2H Gold/pGBKT7-CPI2 | MATa, trp1-901, leu2-3, 112, ura3-52, his3-200 pGBKT7-CPI2 | This study |
| Y2H Gold/pGBKT7-CPI3 | MATa, trp1-901, leu2-3, 112, ura3-52, his3-200 pGBKT7-CPI3 | This study |
| Y2H Gold/pGBKT7-CPI4 | MATa, trp1-901, leu2-3, 112, ura3-52, his3-200 pGBKT7-CPI4 | This study |
| Y2H Gold/pGBKT7-CPI5 | MATa, trp1-901, leu2-3, 112, ura3-52, his3-200 pGBKT7-CPI5 | This study |
| Y2H Gold/pGBKT7-CPI6 | MATa, trp1-901, leu2-3, 112, ura3-52, his3-200 pGBKT7-CPI6 | This study |
| Y2H Gold/pGBKT7-CPI7 | MATa, trp1-901, leu2-3, 112, ura3-52, his3-200 pGBKT7-CPI7 | This study |
| Y2H Gold/pGBKT7-CPI8 | MATa, trp1-901, leu2-3, 112, ura3-52, his3-200 pGBKT7-CPI8 | This study |
| Y2H Gold/pGBKT7-CPI9 | MATa, trp1-901, leu2-3, 112, ura3-52, his3-200 pGBKT7-CPI9 | This study |
| Y2H Gold/pGBKT7-CPI10 | MATa, trp1-901, leu2-3, 112, ura3-52, his3-200 pGBKT7-CPI10 | This study |
| Y2H Gold/pGBKT7-53 | MATa, trp1-901, leu2-3, 112, ura3-52, his3-200 pGBKT7-53 | This study |
| Y2H Gold/pGBKT7-Lam | MATa, trp1-901, leu2-3, 112, ura3-52, his3-200 pGBKT7-Lam | This study |
| Y187/pGADT7 | MATα, ura3-52, his3-200, ade2-101, trp1-901, leu2-3, 112, met-, pGADT7 | This study |
| Y187/pGADT7-CoTem1_96-302_ | MATα, ura3-52, his3-200, ade2-101, trp1-901, leu2-3, 112, met-, pGADT7-CoTem1-96-302 | This study |
| Y187/pGADT7-CoTem1_96-302_^T146A^ | MATα, ura3-52, his3-200, ade2-101, trp1-901, leu2-3, 112, met-, pGADT7-CoTem1-96-302-T146A | This study |
| Y187/pGADT7-T | MATα, ura3-52, his3-200, ade2-101, trp1-901, leu2-3, 112, met-, pGADT7-T | This study |

**TABLE S2B** Primers used in this study.

| Primer Name | Sequence (5' → 3') | Used for |
| --- | --- | --- |
| pGD_F1 | CTCGAGCTGCAGATGAATCGTAGATACTGAAAAAC | Y2H vector pGADT7 |
| pGD_R1 | GAATTCACTGGCCTCCATGGCCATATGAGCGTAAT |  |
| Y_Tem1_F2 | CATGGAGGCCGAATTCCGCAACCACGTCGTCATCA | Y2H vector pGADT7-CoTem196-302 |
| Y_Tem1_R1 | GCAGGTCGACGGATCCTCAGCAAGACTGGTACAGC |  |
| Tem1_T146A_F1 | CGAGGACTACATCCAGGCACTGGGCGTCAACTTTA | Y2H vector pGADT7-CoTem196-302T146A vector |
| Tem1_T146A_R1 | TAAAGTTGACGCCCAGTGCCTGGATGTAGTCCTCG |  |
| pGB_F1 | GGATCCGTCGACCTGCAGCGGCCGCATAACTAGCA | Y2H vector pGBKT7 |
| pGB_R1 | GAATTCGGCCTCCATGGCCATATGCAGGTCCTCCT |  |
| Y_CPI1_BD_F1 | GGAGGCCAGTGAATTCTCGCGCCACCAAGCAGTTC | Y2H vector pGBKT7-CPI1 |
| Y_CPI1_BD_R1 | TCATCTGCAGCTCGAGCTATTCGAGCAGTCCAGCA |  |
| Y_CPI2_BD_F1 | GGAGGCCAGTGAATTCGACTCCGGAAAGACTACCA | Y2H vector pGBKT7-CPI2 |
| Y_CPI2_BD_R1 | TCATCTGCAGCTCGAGTTACGACTTGTTGAACAGA |  |
| Y_CPI3_BD_F1 | GGAGGCCAGTGAATTCAGCTTTTCCAACTCTCGCC | Y2H vector pGBKT7-CPI3 |
| Y_CPI3_BD_R1 | TCATCTGCAGCTCGAGTTACCTGAAGCGCGCTCTG |  |
| Y_CPI4_BD_F1 | GGAGGCCAGTGAATTCAGCCAAATTTTGACCTCTC | Y2H vector pGBKT7-CPI4 |
| Y_CPI4_BD_R1 | TCATCTGCAGCTCGAGTCAGTTGGCAAAGATCCGC |  |
| Y_CPI5_BD_F1 | GGAGGCCAGTGAATTCAAGTTCTTTCTTCCCCTCG | Y2H vector pGBKT7-CPI5 |
| Y_CPI5_BD_R1 | TCATCTGCAGCTCGAGTTAGAGAACGGCGGCGACG |  |
| Y_CPI6_BD_F1 | GGAGGCCAGTGAATTCAGGTTCGCCACGGCTGTCA | Y2H vector pGBKT7-CPI6 |
| Y_CPI6_BD_R1 | TCATCTGCAGCTCGAGTCAAAGCTCGAGATTGAAG |  |
| Y_CPI7_BD_F1 | GGAGGCCAGTGAATTCTTCAAGTGGGCGCAACAAA | Y2H vector pGBKT7-CPI7 |
| Y_CPI7_BD_R1 | TCATCTGCAGCTCGAGCTATTCGGAAATGAACGTG |  |
| Y_CPI8_BD_F1 | GGAGGCCAGTGAATTCTCGCCCACGTACACCATGT | Y2H vector pGBKT7-CPI8 |
| Y_CPI8_BD_R1 | TCATCTGCAGCTCGAGCTACTTGGACGACCTCCAC |  |
| Y_CPI9_BD_F1 | GGAGGCCAGTGAATTCGATCGCATCAAGGAGAAGA | Y2H vector pGBKT7-CPI9 |
| Y_CPI9_BD_R1 | TCATCTGCAGCTCGAGCTAGATGTTGCCAATCTCC |  |
| Y_CPI10_BD_F1 | GGAGGCCAGTGAATTCGCACAACAACAGACTTTTG | Y2H vector pGBKT7-CPI10 |
| Y_CPI10_BD_R1 | TCATCTGCAGCTCGAGTCATGACCAGAACTGGTTA |  |
| CPI1_KO_F1 | TCCCTTAATTCTCCGTCTGCACGTTTTCATGCTTC | CPI1 gene deletion vector pBI-CPI1-BH |
| CPI1_KO_R1 | CAATCTGATCATGAGATCTCGTCGTCTTGCGACTT |  |
| CPI1_HPH_F1 | GCCGACCGGGAACCAGATCGATTGACGGAGGAATG |  |
| CPI1_HPH_R1 | GCTCCTTCAATATCAAAAAACATCAGGCGAAAGGA |  |
| CPI1_KO_F2 | GATTGCACAAAAGGCCAGATTTCACCTCAAGCGGC | Confirmation of targeted gene deletion of CPI1 |
| CPI1_KO_R2 | GTTTCGACGAGATCGAGGAGCAAGTCACAGGGTTC |  |
| CPI2_KO_F1 | TCCCTTAATTCTCCGGGAGGATCTCAACCCACGTA | CPI2 gene deletion vector pBI-CPI2-BH |
| CPI2_KO_R1 | CAATCTGATCATGAGCTCCAACTGTCCCTCTCCTG |  |
| CPI2_HPH_F1 | GCCGACCGGGAACCATGTGGCGTATGTTGTGAGAT |  |
| CPI2_HPH_R1 | GCTCCTTCAATATCACGATAATGCAGTTGGACTCG |  |
| CPI2_KO_F2 | CCCACATGGAGATACGGTCTCCCGGACACGGCCCG | Confirmation of targeted gene deletion of CPI2 |
| CPI2_KO_R2 | TCCAGGGTCTTATGGACCAAAAGCGTCATGACGGC |  |
| CPI3_KO_F1 | TCCCTTAATTCTCCGCATCAAATCCATCAGGCTCT | CPI3 gene deletion vector pBI-CPI3-BH |
| CPI3_KO_R1 | CAATCTGATCATGAGTAAAGCTACTCCATACGCCT |  |
| CPI3_HPH_F1 | GCCGACCGGGAACCATTGTTACCCTCACCTCGGAT |  |
| CPI3_HPH_R1 | GCTCCTTCAATATCAAGTTGGCCGAATAGCTCGGT |  |
| pBIG4MR(BS)revF1 | CTCATGATCAGATTGTCGTTTCCCGCCTTCAGTTT | pBI-G4MRBrev Binary vector |
| pBIG4MR(BS)revR1 | CGGAGAATTAAGGGAGTCACGTTATGACCTCTAGT |  |
| HPH_F1A | TGATATTGAAGGAGCATTTTTTGGGCTTGGCTGGA | Hygromycin-resistance cassette |
| HPH_R1B | TGGTTCCCGGTCGGCAGCGAAAGCGAGAGGGTTGG |  |
| CoNPC1_L_F1 | TCGCTATTACGCCAGACTCAGCACTCGGTACCTCC | CoNPC1 gene deletion vector pPZP-CoNPC1-N |
| CoNPC1_L_R2 | CTGTGGCGTTGGCACTCTTTCCTTGTCGAAGTCCC |  |
| CoNPC1_R_F2 | AGTTGATAATGGGAAACAAAGCTTCTGGGAGCTCC |  |
| CoNPC1_R_R1 | GATTCATTAATGCAGTGCTTATGGGAGTTTCGACG |  |
| CoNPC1_KO_F2 | ACCTACCTACCTAGGTACCTACGCTAAGCACTGGC | Confirmation of targeted gene deletion of CoNPC1 |
| CoNPC1_KO_R2 | AGATGGGTCATACCTCATCCCTTACTTCAACGACC |  |
| Neo_F1 | GTGCCAACGCCACAGTGCCCCACATCTCCCGGCTG | Neomycin-resistance cassette |
| Neo_R1 | TTCCCATTATCAACTCAGAAGAACTCGTCAAGAAG |  |
| glyGFPF1 | GGAGGAGGAGGAGGAATGGTGAGCAAGGGC | glyGFP |
| GFPR1 | TTACTTGTACAGCTCGTCCATGCCGAGAGT |  |
| mCherry_F1-3 | TTAAGATCTGTACAGCTCGTCCATGCCGCCGGTGG | glymCherry |
| 40glymCherry_R1 | GGTGGTGGTGGTGGTATGGTGAGCAAGGGCGAGGAGGATA |  |
| CoNPC1_com_F1 | TCCCTTAATTCTCCG AGCTGTTTCAACTCGACCTA | pBI-CoNPC1-GFP-S plasmid |
| CoNPC1_com_R1 | CAATCTGATCATGAG TTGACAATGCTGTCGAGGTC |  |
| CoNPC1_GFP_F1 | CGAGCTGTACAAGTAAACGCGGAAAAAAAAATTGCAATGA |  |
| CoNPC1_GFP_R1 | TTCCTCCTCCTCCTCCGTAGTCGTCGTCCGAGTCAGTGTC |  |
| CoNPC2_mCherry_F1-3 | CTGTACAGATCTTAAGTGATCGACCAACGCATCAC | pBI-CoNPC2-mCherry-B plasmid |
| CoNPC2_mCherry_R1 | ACCACCACCACCACCAAGCTCGAGATTGAAGAAAC |  |
| CoRAB7_com_F1 | TCCCTTAATTCTCCGCATCAACTCATGCTGGGCCG | pBI-CoRAB7-GFP-S plasmid |
| CoRAB7_com_R1 | CAATCTGATCATGAGCCTTCCGGAATTACCCAGTG |  |
| CoRAB7_GFP_F1 | CGAGCTGTACAAGTAAGGAGCTGCAATGAGGACATG |  |
| CoRAB7_GFP_R1 | TTCCTCCTCCTCCTCCGCAGGCGCAGCCGTCGCGCG |  |
| CoVPH1_com_F1 | TCGCTATTACGCCAGTCTAGATCCTTGTCCACGGC | pBI-CoVPH1-mCherry-S plasmid |
| CoVPH1_com_R1 | GATTCATTAATGCAGATGGGTCCTTTCTGTCCGAC |  |
| CoVPH1_mCherry_F1-3 | CTGTACAGATCTTAAGGACTCTTTCAGCTGGTAGC |  |
| CoVPH1_mCherry_R1 | ACCACCACCACCACCACCCAGGTACTCCTTCAGCT |  |
| pCAMSUR_F2 | TGTGCTGGGGCCGCGCTGGTGGCGTGCGTATTGGCCTGGA | pCAMSUR-TEF Binary vector |
| 40TEFpF2 | GTTTGACGGTGATGTATGGAAGATGGAGTGAAGTACGGTT |  |
| CoNPC1_OX_F1 | ACATCACCGTCAAACATGAGTATGCGAACGCTGCT | pCAMSUR-TEF-CoNPC1-3MYC plasmid |
| CoNPC1_OX_R1 | CGCGGCCCCAGCACAAGCTGTTTCAACTCGACCTA |  |
| CoNPC1_Myc_F1 | GGCGGAGGCGGAGGCGAGCAGAAGCTCATCTCCGAGGAGGACCTCGAGCAGAAGCTCATCTCCGAGGAGGACCTCGAGCAGAAGCTCATCTCCGAGGAGGACCTCTAGACGCGGAAAAAAAAATTGCA |  |
| CoNPC1_Myc_R1 | GCCTCCGCCTCCGCCGTAGTCGTCGTCCGAGTCAGTGTCC |  |
| CoNPC2_OX_R1 | CGCGGCCCCAGCACAACTGCCTTAGCGACATGGAC | pCAMSUR-TEF-CoNPC2-3MYC plasmid |
| CoNPC2_OX_F1 | ACATCACCGTCAAACATGAGGTTCGCCACGGCTGT |  |
| CoNPC2_Myc_F1 | GGCGGAGGCGGAGGCGAGCAGAAGCTCATCTCCGAGGAGGACCTCGAGCAGAAGCTCATCTCCGAGGAGGACCTCGAGCAGAAGCTCATCTCCGAGGAGGACCTCTAGGTGATCGACCAACGCATCAC |  |
| CoNPC2_Myc_R1 | GCCTCCGCCTCCGCCAAGCTCGAGATTGAAGAAAC |  |
